# Preclinical Evaluation of a New Series of Albumin-Binding ^177^Lu-Labeled PSMA-Based Low-Molecular-Weight Radiotherapeutics

**DOI:** 10.3390/molecules28166158

**Published:** 2023-08-21

**Authors:** Srikanth Boinapally, Suresh Alati, Zirui Jiang, Yu Yan, Alla Lisok, Rajan Singh, Gabriela Lofland, Il Minn, Robert F. Hobbs, Martin G. Pomper, Sangeeta Ray Banerjee

**Affiliations:** 1 Russell H. Morgan Department of Radiology and Radiological Science, 1550 Orleans Street, Cancer Research Building 2, Baltimore, MD 21287, USA; sboinap1@gmail.com (S.B.); salati2@jhmi.edu (S.A.); zjiang40@jhmi.edu (Z.J.); iminn1@jhmi.edu (I.M.); rhobbs3@jhmi.edu (R.F.H.); mpomper@jhmi.edu (M.G.P.); 2 Sidney Kimmel Comprehensive Cancer Center, Johns Hopkins University, Baltimore, MD 21287, USA

**Keywords:** prostate-specific membrane antigen, radiopharmaceutical therapy, albumin, linker, prostate cancer

## Abstract

Prostate-specific membrane antigen (PSMA)-based low-molecular-weight agents using beta(β)-particle-emitting radiopharmaceuticals is a new treatment paradigm for patients with metastatic castration-resistant prostate cancer. Although results have been encouraging, there is a need to improve the tumor residence time of current PSMA-based radiotherapeutics. Albumin-binding moieties have been used strategically to enhance the tumor uptake and retention of existing PSMA-based investigational agents. Previously, we developed a series of PSMA-based, β-particle-emitting, low-molecular-weight compounds. From this series, ^177^Lu-L1 was selected as the lead agent because of its reduced off-target radiotoxicity in preclinical studies. The ligand L1 contains a PSMA-targeting Lys-Glu urea moiety with an N-bromobenzyl substituent in the ε-amino group of Lys. Here, we structurally modified ^177^Lu-L1 to improve tumor targeting using two known albumin-binding moieties, 4-(*p*-iodophenyl) butyric acid moiety (IPBA) and ibuprofen (IBU), and evaluated the effects of linker length and composition. Six structurally related PSMA-targeting ligands (Alb-L1–Alb-L6) were synthesized based on the structure of ^177^Lu-L1. The ligands were assessed for in vitro binding affinity and were radiolabeled with ^177^Lu following standard protocols. All ^177^Lu-labeled analogs were studied in cell uptake and selected cell efficacy studies. In vivo pharmacokinetics were investigated by conducting tissue biodistribution studies for ^177^Lu-Alb-L2–^177^Lu-Alb-L6 (2 h, 24 h, 72 h, and 192 h) in male NSG mice bearing human PSMA+ PC3 PIP and PSMA− PC3 flu xenografts. Preliminary therapeutic ratios of the agents were estimated from the area under the curve (AUC_0-192h_) of the tumors, blood, and kidney uptake values. Compounds were obtained in >98% radiochemical yields and >99% purity. PSMA inhibition constants (*K*_i_s) of the ligands were in the ≤10 nM range. The long-linker-based agents, ^177^Lu-Alb-L4 and ^177^Lu-Alb-L5, displayed significantly higher tumor uptake and retention (*p* < 0.001) than the short-linker-bearing ^177^Lu-Alb-L2 and ^177^Lu-Alb-L3 and a long polyethylene glycol (PEG) linker-bearing agent, ^177^Lu-Alb-L6. The area under the curve (AUC_0-192h_) of the PSMA+ PC3 PIP tumor uptake of ^177^Lu-Alb-L4 and ^177^Lu-Alb-L5 were >4-fold higher than ^177^Lu-Alb-L2, ^177^Lu-Alb-L3, and ^177^Lu-Alb-L6, respectively. Also, the PSMA+ PIP tumor uptake (AUC_0-192h_) of ^177^Lu-Alb-L2 and ^177^Lu-Alb-L3 was ~1.5-fold higher than ^177^Lu-Alb-L6. However, the lowest blood AUC_0-192h_ and kidney AUC_0-192h_ were associated with ^177^Lu-Alb-L6 from the series. Consequently, ^177^Lu-Alb-L6 displayed the highest ratios of AUC(tumor)-to-AUC(blood) and AUC(tumor)-to-AUC(kidney) values from the series. Among the other agents, ^177^Lu-Alb-L4 demonstrated a nearly similar ratio of AUC(tumor)-to-AUC(blood) as ^177^Lu-Alb-L6. The tumor-to-blood ratio was the dose-limiting therapeutic ratio for all of the compounds. Conclusions: ^177^Lu-Alb-L4 and ^177^Lu-Alb-L6 showed high tumor uptake in PSMA+ tumors and tumor-to-blood ratios. The data suggest that linker length and composition can be modulated to generate an optimized therapeutic agent.

## 1. Introduction

Prostate cancer is the most commonly diagnosed non-cutaneous cancer among men in the United States and globally [1]. Death from prostate cancer occurs mainly in patients with aggressive, androgen-insensitive, metastatic disease [2,3]. Prostate-specific membrane antigen (PSMA) is a tumor-associated antigen overexpressed in prostate adenocarcinoma cells, regardless of androgen status, in the neovasculature of solid tumors, and has a low expression in benign and extra-prostatic tissues [4,5]. Studies have shown that radiopharmaceutical therapy targeting PSMA with β-particle-emitting ^177^Lu (half-life 6.7 d) is a life-prolonging treatment option for patients with metastatic castration-resistant prostate cancer [6]. Recently, a low-molecular-weight agent, ^177^Lu-PSMA-617, demonstrated a low toxicity profile compared to the standard of care and received regulatory approval [6,7]. Although highly promising, the median overall survival among randomized patients was 15.3 months in the treatment vs. 11.3 months in the control group, suggesting that further improvements are needed [6,8]. One way to achieve this is through the pharmacokinetic optimization of agents similar to ^177^Lu-PSMA-617.

**Figure 1:**
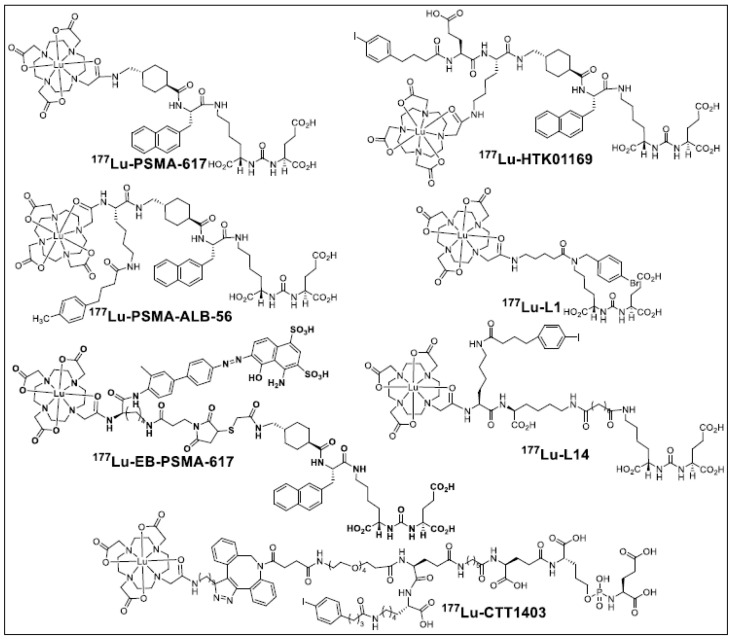
Structures of clinically studied PSMA-targeted albumin-binding agents (^177^Lu-PSMA-ALB-56, ^177^Lu-EB-PSMA-617, ^177^Lu-CTT1403), ^177^Lu-PSMA-617, PSMA I&T, ^177^Lu-L1, and ^177^Lu-L14.

Several approaches involving the structural manipulation of PSMA-targeting ureas are currently under intense investigation to improve the efficacy of ^177^Lu-PSMA-617 [8]. Examples of such optimization include the development of bivalent or high-affinity agents and the incorporation of pharmacophores to enhance their blood circulation [9,10,11,12,13,14]. Along this direction, incorporating an albumin-binding moiety into PSMA-based, low-molecular-weight agents has gained significant attention [11,13,15,16,17,18,19,20,21]. Well-characterized, small-molecule moieties (e.g., 4-(*para*-iodophenyl) butyric acid (IPBA), truncated Evans blue, ibuprofen (IBU), and fatty acids), referred to as “blood half-life extenders”, non-covalently bind to serum albumin and have been utilized extensively [22]. Several agents of this class have entered clinical trials, as shown in Figure 1.

The majority of these agents are derived from the structure of PSMA-617 [20,23,24]. Preclinical and clinical data have revealed increased tumor targeting of such agents. However, increased radiation dose delivery to the salivary glands and kidneys was also observed partly due to PSMA expression in these tissues [13,18,23]. In addition, increased off-target radiotoxicity, specifically hematologic toxicity, has been demonstrated in clinical studies [13,23]. Accordingly, current preclinical efforts are focused on optimizing the PSMA-targeting moiety [20,25], linker composition [12,26], and albumin-binding motif [20,26] to maximize the tumor-to-kidney radiation dose ratios. Improving tumor targeting and weakening albumin binding to reduce blood, kidney, or salivary gland uptake is the primary rationale for newly developed agents [18,25,26].

**Figure 2:**
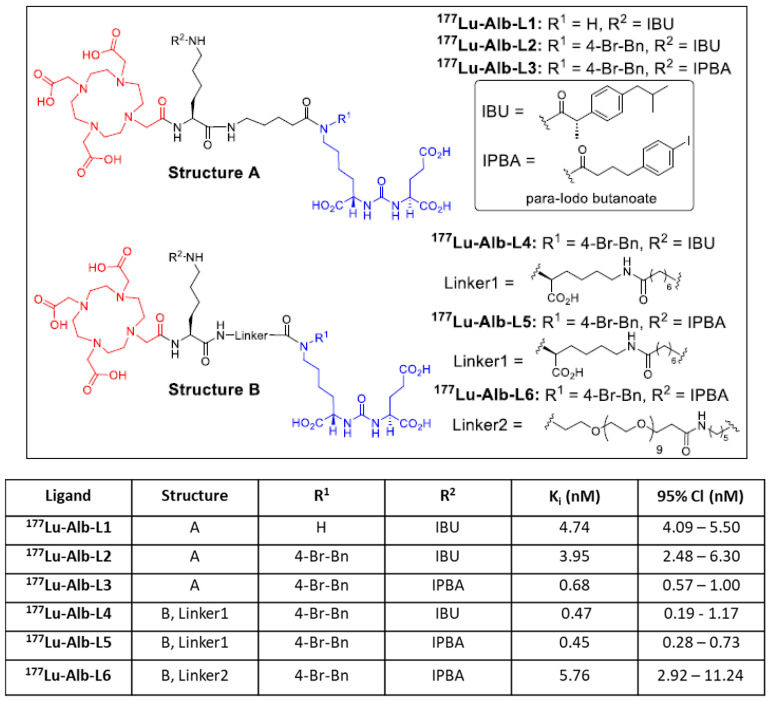
Structures of new agents studied in this report are derived from Structure A (Alb-L1, Alb-L2, and Alb-L3) and Structure B (Alb-L4, Alb-L5, and Alb-L6).

We recently developed a new series of PSMA-based agents with reduced off-target toxicity, and one of which was translated into the clinic [27]. The lead agent, L1, was also investigated using alpha (α)-particle-emitting radionuclides, including ^225^Ac, ^213^Bi, ^212^Pb, and ^211^At [28,29,30]. In the current study, we evaluated six albumin-binding agents by conjugating IBU and IPBA using two bifunctional linkers. Our primary goal was to generate an agent with enhanced tumor uptake and retention compared to ^177^Lu-L1. We investigated two albumin-binding moieties and 1,4,7,10-tetraazacyclododecane-1,4,7,10-tetraacetic acid mono-amide (DOTA-monoamide) as a chelating unit to generate six ligands bearing either a short (A) (^177^Lu-Alb-L1–^177^Lu-Alb-L3) or long linker (B) (^177^Lu-Alb-L4–Alb-L6) (Figure 2). Our data revealed that the linker length and composition might be critical in optimizing PSMA− and albumin-based agents for clinical use.

## 2. Results

### 2.1. Synthesis and Binding Affinities of Ligands Alb-L1 to Alb-L6

We designed and synthesized six ligands using (i) PSMA-targeting Glu-Lys (N-*p*-bromo-benzyl) urea moiety, (ii) short linker (Structure A) or long linker (Structure B), and (iii) albumin-binding moieties IPBA and IBU, as shown in Figure 2. Standard solution-phase peptide conjugation chemistry was employed to synthesize these new compounds, as detailed in the Supplementary Materials. After evaluating ^177^Lu-Alb-L1 to ^177^Lu-Alb-L5, we studied ^177^Lu-Alb-L6, containing a long polyethylene glycol (PEG) linker, to reduce the blood uptake of these agents.

### 2.2. In Vitro Characterization 

#### Binding Affinity, Cell Uptake, and Internalization

**Figure 3:**
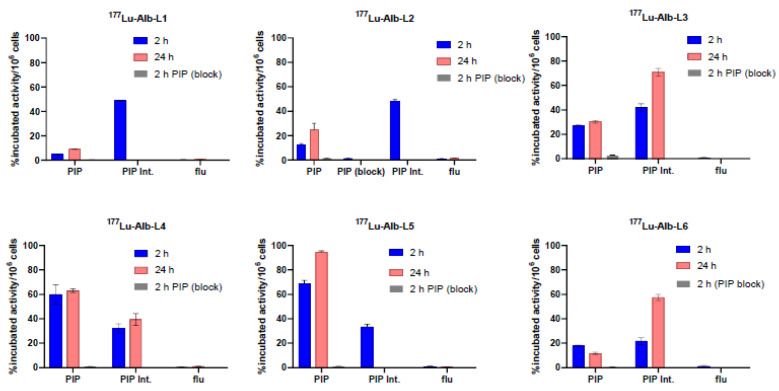
Cell uptake and internalization (mean ± SD, n = 3) of ^177^Lu-Alb-L1–^177^Lu-Alb-L6 in PSMA+ PC3 PIP cells and PSMA− PC3 flu cells (~1 million) at 37 °C. PSMA expression blockade studies in PSMA+ PC3 PIP cells of ^177^Lu-Alb-L1–^177^Lu-Alb-L6 were performed at 2 h post-incubation. The cells (~1 million) were pre-incubated with a known PSMA inhibitor, ZJ43 (10 µM final concentration), for 30 min before the corresponding radioactive dose was added to the medium.

The new ligands displayed high binding affinity to PSMA with *K*_i_ values ranging from 0.12 nM to 11.24 nM, as listed in Figure 2. Ligands, Alb-L4, and Alb-L5 from Structure B construct and Alb-L3 displayed significantly higher affinity (*p* < 0.05) than the rest of the ligands. The albumin-binding properties of the agents revealed a substantially higher affinity for the long-linker-based agents, ^177^Lu-Alb-L4 and ^177^Lu-Alb-L5, compared to ^177^Lu-Alb-L6 and the short-linker-based ^177^Lu-Alb-L2, as well as ^177^Lu-Alb-L3 (Supplementary Table S3). The cell uptake and internalized fraction of the ^177^Lu-labeled agents are shown in Figure 3. Except for ^177^Lu-Alb-L1 (without a *p*-bromo-benzyl group on the PSMA-targeting unit), all of the agents demonstrated high cell uptake in the PSMA+ PC3 PIP cells, i.e., in the range of 28% to 75% of the incubated dose at 2 h, and this was slightly increased up to 30% to 77% at 24 h incubation. From the series, ^177^Lu-Alb-L4 and ^177^Lu-Alb-L5 displayed high uptake, 60% and 75%, respectively, at 2 h, and 63% and 77% at 24 h of incubation in PSMA+ PC3 PIP cells. The percentage of internalization for the agents in PSMA+ PC3 PIP cells was in the range of 32–47% at 2 h. The uptake of the radioligands in the PSMA− PC3 flu cells was significantly lower (~100-fold) than that of the PSMA+ PC3 PIP cells, revealing the PSMA-specific binding of the agents. A PSMA expression blocking study was performed using an excess of ZJ43 [31], a known PSMA inhibitor, which caused a significantly low uptake of the radiolabeled agents in PSMA+ PC3 PIP cells (Figure 3). The data further confirmed the PSMA-mediated uptake of ^177^Lu-Alb-L1–^177^Lu-Alb-L6 in PSMA+ PC3 PIP cells.

### 2.3. Clonogenic Survival Assay

**Figure 4:**
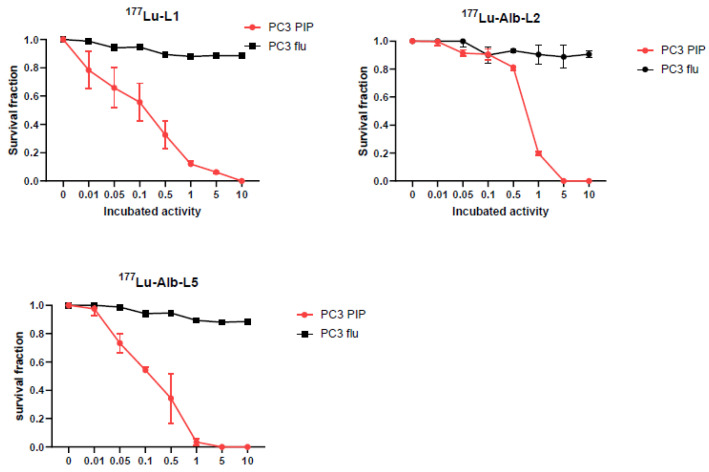
In vitro clonogenic assay. Clonogenic survival of PSMA+ PC3 PIP cells and PSMAPC3 flu cells treated with increasing concentrations of ^177^Lu-Alb-L2, ^177^Lu-Alb-L5, and ^177^Lu-L1 for 48 h at 37 °C.

Clonogenic assays were performed to assess the cellular efficacy of ^177^Lu-Alb-L2 and ^177^Lu-Alb-L5 and were compared with ^177^Lu-L1, as shown in Figure 4. The data revealed a decrease in the survival of PSMA+ PC3 PIP cells with increasing concentrations of radioactivity, with complete cell killing reached at a concentration of 370 kBq/mL for ^177^Lu-L1, 185 kBq/mL for ^177^Lu-Alb-L2, and 37 kBq/mL for ^177^Lu-Alb-L5, respectively. The activity to reduce cell survival to 37% (A_0_) was 18.5 kBq/mL for ^177^Lu-L1, ~20 kBq/mL for ^177^Lu-Alb-L2, and ~10 kBq/mL for ^177^Lu-Alb-L5, respectively.

### 2.4. Biodistribution 

Tissue biodistribution studies of ^177^Lu-Alb-L2–^177^Lu-Alb-L6 were performed at 2 h, 24 h, 48 h, and 192 h after injection to evaluate the tumor targeting and clearance of the agents from blood and normal tissues. The biodistribution of ^177^Lu-L1 was conducted at 24 h post-injection for the agents for direct comparison. The biodistribution studies of ^177^Lu-Alb-L2 and ^177^Lu-Alb-L5, as well as ^177^Lu-L1 (24 h), were performed in one experiment to keep the experimental variations minimal (for example, tumor size, animals’ age, and specific activity of ^177^Lu). Similarly, biodistribution studies of ^177^Lu-Alb-L3 and ^177^Lu-Alb-L4, as well as ^177^Lu-L1 (24 h), were conducted head-to-head in one experiment. The biodistribution study of ^177^Lu-Alb-L6 was performed separately using tumors of a similar size.

**Figure 5:**
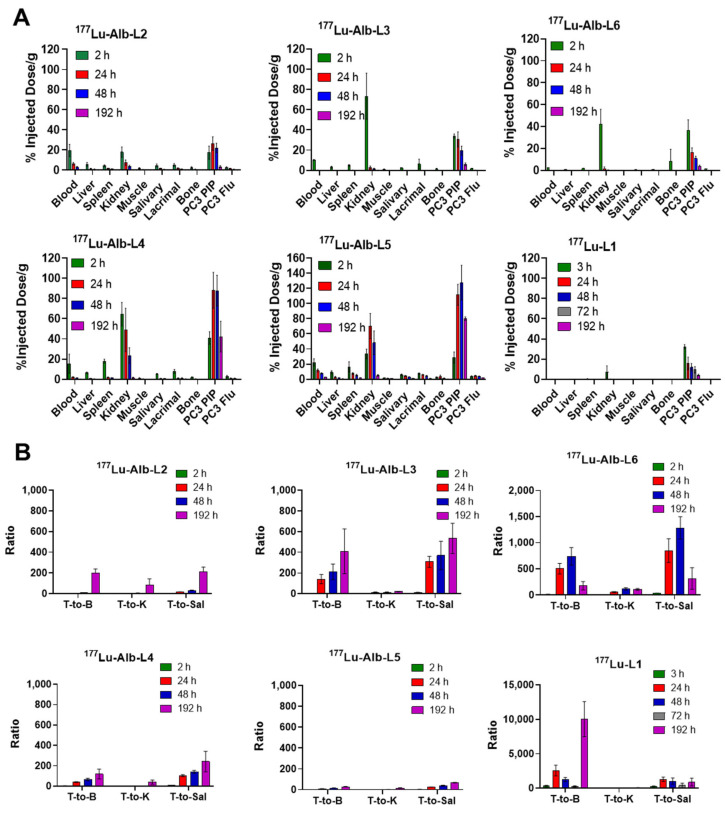
(**A**) Tissue biodistribution data of ^177^Lu-Alb-L2–^177^Lu-Alb-L6 and ^177^Lu-L1 are shown as the percentage of injected dose per gram of tissue, mean ± SD, n = 3–4 mice, dose: 1.87 MBq (intravenous injection via tail-vein), tumor model: PSMA+ PC3 PIP, and PSMA− PC3 flu tumor-bearing male NSG mice. (**B**) The tumor-to-blood (T/B), tumor-to-kidney (T/K), and tumor-to-salivary (T/Sal) ratios were obtained from the biodistribution data of ^177^Lu-L1 and ^177^Lu-Alb-L2–^177^Lu-Alb-L6. Biodistribution data of ^177^Lu-L1 were obtained from [27].

The results of the new agents and ^177^Lu-L1 are presented in Figure 5 and Supplementary Tables S4–S10. Biodistribution data of ^177^Lu-L1 (3–192 h) were acquired from our previously reported studies [27]. A fast tumor accumulation was observed for ^177^Lu-Alb-L2, which reached 17.45 ± 6.51%ID/g at 2 h post-injection and displayed higher uptake, 26.41 ± 6.73%ID/g at 24 h and 22.00 ± 4.71%ID/g at 48 h, respectively. At 192 h post-injection, tumor uptake was low, 3.39 ± 1.03%ID/g. ^177^Lu-Alb-L3 displayed similar pharmacokinetics as ^177^Lu-Alb-L2, 34.05 ± 1.98%ID/g at 2 h, 30.55 ± 7.44%ID/g at 24 h, 19.61 ± 4.46%ID/g at 48 h, and 5.94 ± 1.38%ID/g at 192 h. 

The long-linker-based agents (Structure B), ^177^Lu-Alb-L4 and ^177^Lu-Alb-L5, displayed significantly higher tumor uptake and retention than ^177^Lu-Alb-L2 and ^177^Lu-Alb-L3. The tumor uptake of ^177^Lu-Alb-L4 was 40.89 ± 4.73%ID/g at 2 h, 88.04 ± 16.51%ID/g at 24 h, 87.74 ± 14.09%ID/g at 48 h, and 42.22 ± 14.05%ID/g at 192 h, respectively. For ^177^Lu-Alb-L5, it was 28.78 ± 7.25%ID/g at 2 h, 111.47 ± 13.85%ID/g at 24 h, 127.44 ± 22.85%ID/g at 48 h, and 70.96 ± 2.34%ID/g at 192 h, respectively. ^177^Lu-Alb-L6 displayed moderate uptake and fast clearance: 38.73 ± 1.26%ID/g at 2 h, 13.72 ± 3.55%ID/g at 24 h, 9.62 ± 0.96%ID/g at 48 h, and 2.22 ± 0.37%ID/g at 192 h. The tumor uptake values of ^177^Lu-L1 at 24 h were 12.86 ± 0.98–14.47 ± 1.32%ID/g. The tumor uptake and clearance of ^177^Lu-L1 were in the same range as ^177^Lu-Alb-L6 (30.81 ± 2.86%ID/g, 15.67 ± 6.25%ID/g, 11.94 ± 3.83%ID/g, 9.33 ± 3.18%ID/g, and 4.21 ± 1.45%ID/g at 192 h). The uptake in the PSMA− PC3 flu tumors was low for the agents. The highest uptake in the PSMA− PC3 flu tumor was associated with ^177^Lu-Alb-L5, 3.87 ± 0.70%ID/g at 2 h, 4.55 ± 0.76%ID/g at 24 h, 3.95 ± 0.50%ID/g at 48 h, and 1.87 ± 0.23%ID/g at 192 h post-injection, respectively.

The blood uptake levels were high at 2 h for ^177^Lu-Alb-L2, with 19.59 ± 6.06%ID/g; ^177^Lu-Alb-L3, with 10.29 ± 0.41%ID/g; ^177^Lu-Alb-L4, with 15.48 ± 7.12%ID/g; ^177^Lu-Alb-L5, with 22.36 ± 4.72%ID/g; and ^177^Lu-Alb-L6, with 2.97 ± 0.45%ID/g, respectively. ^177^Lu-Alb-L6 displayed the fastest clearance from the circulation owing to the long PEG linker, and the uptake was <0.03 ± 0.01%ID/g at 24 h. However, the blood uptake values of ^177^Lu-L1 (0.18 ± 0.16 at 3 h, 0.01 ± 0.01 at 24 h, 0.03 ± 0.03 at 48 h, 0.02 ±0.02 at 72 h, and 0.00 ±0.00 at 192 h) were significantly lower than those of ^177^Lu-Alb-L6 and the new albumin-binding agents at all time points. ^177^Lu-Alb-L5 displayed the slowest clearance from the blood at 192 h post-injection, 2.77 ± 0.19%ID/g, followed by ^177^Lu-Alb-L4 (2.19 ± 0.46%ID/g), ^177^Lu-Alb-L3 (0.24 ± 0.12%ID/g), and ^177^Lu-Alb-L2 (≤0.01%ID/g).

Kidney uptake was relatively high for ^177^Lu-Alb-L5 (33.45 ± 6.41%ID/g at 2 h, 70.23 ± 16.64 at 24%ID/g, 48.17 ± 15.62%ID/g at 48 h, and 5.06 ± 0.99%ID/g at 192 h) and ^177^Lu-PSMA-Alb-L4 (64.44 ± 8.75%ID/g at 2 h, 48.81 ± 19.86%ID/g at 24 h, 23.46 ± 7.75%ID/g at 48 h, and 1.34 ± 0.82 at 192 h). ^177^Lu-Alb-L3 displayed the highest kidney uptake at 2 h, 73.28 ± 22.85%ID/g, and the fastest clearance, 2.77 ± 1.56%ID/g at 24 h. ^177^Lu-Alb-L2 and ^177^Lu-Alb-L6 displayed low kidney uptake of ~17.86%ID/g at 2 h. ^177^Lu-Alb-L6 displayed fast renal clearance resulting in <1%ID/g after 24 h; in contrast, ^177^Lu-Alb-L2 showed a slow and steady wash-out from the kidneys from ~7.44 ± 2.64% IA/g at 24 h post-injection to 3.74 ± 1.05%ID/g at 48 h post-injection. The kidney uptake and clearance values of ^177^Lu-L1 were in the range of ^177^Lu-Alb-L6 (5.16 ± 2.38%ID/g at 3 h, 0.28 ± 0.18%ID/g at 24 h, 0.22 ± 0.10%ID/g at 72 h, and 0.01 ± 0.00%ID/g at 192 h).

The activity levels in the salivary and lacrimal glands of the agents were comparable to the blood levels and were relatively high for ^177^Lu-Alb-L5 (salivary glands, 5.83 ± 1.06%ID/g at 2 h, 4.35 ± 0.66%ID/g at 24 h, 3.20 ± 0.24 h %ID/g at 48 h, and 1.18 ± 0.09%ID/g at 192 h) and ^177^Lu-Alb-L4 (salivary glands, 5.40 ± 0.33%ID/g at 2 h, 0.86 ± 0.21%ID/g at 24 h, 0.62 ± 0.07%D/g at 48 h, and 0.18 ± 0.06%ID/g at 192 h). Similarly, the uptake of the other normal tissues, including the lung and liver, were high for ^177^Lu-Alb-L5 and decreased continuously over time. For the agents ^177^Lu-Alb-L2, ^177^Lu-Alb-L3, ^177^Lu-Alb-L4, and ^177^Lu-Alb-L6, normal tissue clearance was below the blood levels and mostly ≤2%ID/g after 24 h.

The selected tumor-to-normal tissue ratios of the agents are shown in Figure 5B. A high blood pool was observed for all of the agents at 2 h after injection, resulting in a low tumor-to-blood ratio (1.0 ± 0.2), and it increased over time. The tumor-to-blood ratios were ~4–8 during 24 h and 48 h post-injection for ^177^Lu-Alb-L2 and ~200–220 after 192 h injection. In contrast, ^177^Lu-Alb-L3 displayed fast blood clearance, ~200–250 at 24–48 h, and remained in that range until 192 h. For ^177^Lu-Alb-L4, the ratios were in the range of ~50 during 24–48 h and ~100–150 at 192 h. The slowest clearance was observed for ^177^Lu-Alb-L5; the tumor-to-blood ratios were ~10–20 during 24–48 h and ~30 during 192 h. The tumor-to-blood values were >300 for ^177^Lu-L1 at all time points. 

The tumor-to-kidney ratio was <1 for ^177^Lu-Alb-L2–^177^Lu-Alb-L5 at 2 h and remained at ≤10 during 24–48 h and increased significantly, ~20–50 at 192 h after injection. The lowest tumor-to-kidney ratio was associated with ^177^Lu-Alb-L5. In contrast, the tumor-to-kidney values were >4 for ^177^Lu-L1 at 24 h and remained >50 at all time points. A similar time course was observed for the tumor-to-liver, tumor-to-salivary, and tumor-to-lacrimal glands, as well as the tumor-to-bone ratios.

**Figure 6:**
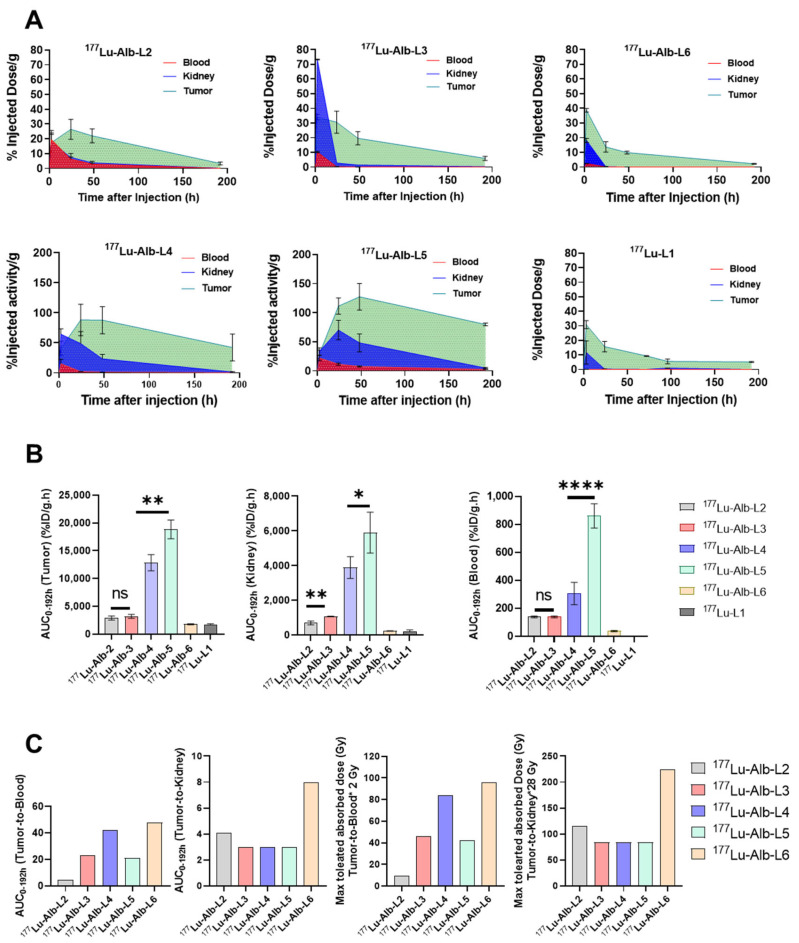
(**A**) Areas under the curves (AUCs) of tumor, blood, and kidney uptakes for ^177^Lu-Alb-L2–^177^Lu-Alb-L6 and ^177^Lu-L1 were calculated. (**B**) AUC values of ^177^Lu-Alb-L2–^177^Lu-Alb-L6 and ^177^Lu-L1 are compared. (**C**) AUC_0-192h_(tumor-to-blood) and AUC_0-192h_(tumor-to-kidney) were analyzed. AUC_0-192h_(tumor-to-blood) is ~867 and AUC_0-192h_(tumor-to-kidney) is ~8, respectively, for ^77^Lu-L1, removed from the graph to provide clarity. The estimated maximum tumor-absorbed doses of ^177^Lu-Alb-L2–^177^Lu-Alb-L6 were analyzed, assuming the maximum absorbed dose for blood 2 Gy and kidney 28 Gy, respectively. *p*-values lower than 0.05 (*p* < 0.05), *p* < 0.01, *p* < 0.001, and *p* < 0.0001 were referred to one (*), two (**), or four (****) asterisks, respectively.

The area under the curve (AUC_0-192h_) values and AUC ratios of tumor-to-blood and tumor-to-kidney over 192 h of the new agents and ^177^Lu-L1 are listed in Figure 6A,B. Tumor AUC_0-192h_ of ^177^Lu-Alb-L5 was significantly higher than ^177^Lu-Alb-L4 (*p* < 0.002). In addition, tumor AUC of ^177^Lu-Alb-L5 and Alb-L4 were ~4-fold higher compared to ^177^Lu-Alb-L2 and ^177^Lu-Alb-L3 and ~6-fold higher than ^177^Lu-Alb-L6 and ^177^Lu-L1. The tumor-to-blood AUC_0-192h_ ratio of ^177^Lu-Alb-L4 was ~2-fold higher than ^177^Lu-Alb-L5, while the tumor-to-kidney AUC_0-192h_ ratios were in the same range. ^177^Lu-Alb-L6 and ^177^Lu-L1 displayed nearly similar tumor AUC_0-192h_ and kidney AUC_0-192h_, and consequently both agents had a similar tumor-to-kidney AUC_0-192h_ (~8). However, the tumor-to-blood AUC_0-192h_ ratios of ^177^Lu-L1 were significantly higher (~867) than the tumor-to-blood AUC_0-192h_ ratios of ^177^Lu-Alb-L6 (~48). The tumor-to-kidney AUC_0-192h_ ratios of ^177^Lu-Alb-L6 were ~2-fold higher than the other albumin-binding agents from the series. The tumor-to-blood AUC_0-192h_ of ^177^Lu-Alb-L6 was comparable to ^177^Lu-Alb-L4; it was ~1.14-fold higher.

## 3. Discussion

Here, we investigated a new series of albumin-binding ^177^Lu-labeled agents based on the structure of ^177^Lu-L1, our previously reported lead agent [27]. As reported by others and us, small-molecule organic moieties with low and reversible binding to serum albumin (Mol wt. 67 kDa) were utilized as a possibility to extend the circulating time of the PSMA-based agents, providing prolonged exposure to the tumors. Such modification significantly increased tumor uptake for the agents, relative to our original compound, ^177^Lu-L1, through an increased blood half-life. These agents were designed and derived from our previously linker-based targeting platform [32,33]. Only two structural features were investigated, linker length and the attachment of two albumin-binding moieties, as shown in Figure 2. Nevertheless, several structure–activity relationships were derived from this small series of compounds, as described below.

We observed significantly higher (*p* < 0.05) binding affinity and PSMA+ cell binding both in vitro and in vivo for new agents, specifically ^177^Lu-Alb-L4 and ^177^Lu-Alb-L5 (Structure B), compared to ^177^Lu-Alb-L2 and ^177^Lu-Alb-L3 (Structure A). ^177^Lu-Alb-L6 displayed low binding affinity, likely due to the long PEG linker, as we noted earlier with a similar construct [34]. Proof-of-concept cell efficacy data further confirmed the effect of enhanced cellular uptake and internalization of ^177^Lu-Alb-L5 (Structure B) compared to ^177^Lu-L1 or ^177^Lu-Alb-L2 (Structure A). Enhanced cellular internalization is critical to radiation-induced DNA damage and PSMA-expressing cancer cell death.

The tumor uptake of ^177^Lu-Alb-L2 and ^177^Lu-Alb-L3 was significantly lower than our previously reported long-linker-based albumin-binding agent, ^177^Lu-L14 (Figure 2) (tumor AUC_0-192h_ of 5740 ± 520%ID/g.h), although ≥1.5-fold higher than ^177^Lu-L1 (tumor AUC_0-192h_ of 1734 ± 130%ID/g.h) (Figure 6A,B) [27]. The long-linker-based agents, ^177^Lu-Alb-L4 (tumor AUC_0-192h_ of 12,857 ± 1469%ID/g.h) and ^177^Lu-Alb-L5 (tumor AUC_0-192h_ of 18,842 ± 1693%ID/g.h) demonstrated a > 2-fold improvement in tumor uptake compared to ^177^Lu-L14. The blood uptake values of ^177^Lu-Alb-L4 (blood AUC_0-192h_ of 307 ± 80) were in the range of ^177^Lu-L14 (blood AUC_0-192h_ of 314 ± 37), indicating a 2-fold improvement in the tumor-to-blood ratios of ^177^Lu-Alb-L4 relative to ^177^Lu-L14. Notably, the kidney uptake of ^177^Lu-Alb-L4 (AUC_0-192h_ of 3895 ± 631) was 1.5-fold higher than ^177^Lu-L14 (AUC_0-192h_ of 2550 ± 347). In contrast, the tumor and PSMA-expressing typical healthy tissue uptake values were significantly lower for ^177^Lu-Alb-L6, most likely owing to low PSMA-binding and albumin-binding properties. Notably, the tumor and kidney AUCs of ^177^Lu-Alb-L6 were in the range of ^177^Lu-L1; however, the blood AUC was ~30-fold higher for ^177^Lu-Alb-L6 than^177^Lu-L1. The in vivo performance of ^177^Lu-Alb-L6 was consistent with the earlier reports, notably, a fast kidney clearance of long PEG-linker-based ^177^Lu-Alb analogs, published by Kelly et al. [11,12].

By comparing the efficacy (in terms of therapeutic index) of the different agents to the absorbed dose thresholds of radiotoxicity for kidneys (~28 Gy) and blood (~2 Gy), the results for the maximum absorbed dose to the tumors of the agents were determined. Figure 6C shows these results. The maximum tumor absorbed dose was estimated to be the lowest of the two values, obtained from the kidney maximum absorbed dose and the blood maximum absorbed dose. For all of the compounds, the dose-limiting organ was found to be the blood. ^177^Lu-Alb-L6, and to a lesser extent, ^177^Lu-Alb-L4, appear to be the more viable compounds with potential tumoricidal absorbed doses able to be delivered to the tumor. 

Renal radiotoxicity has not proved to be a significant issue for ^177^Lu-PSMA-targeted therapy, possibly due to optimal low linear energy transfer radiations of ^177^Lu (β_max_ 0.5 MeV, 1.7 mm). Furthermore, a minimal expression of PSMA has been found in human kidneys. It has been reported that murine PSMA, with 91% similarity to the human PSMA sequence, is overexpressed in the proximal microtubules of the murine renal cortex [35]. The high kidney uptake observed in mice upon the administration of urea-based PSMA therapeutic agents is likely due to the binding to the PSMA mouse isoform. However, most preclinical development of PSMA-based albumin-binding radiotherapeutics has been focused on reducing tumor-to-kidney AUC values [12,20,23]. Furthermore, recent in vitro studies revealed that these albumin-binding compounds are associated with significantly higher binding to human blood plasma than mouse plasma [16,36,37]. Accordingly, tumor-to-blood ratios appeared to be critical in optimizing the albumin-binding agents in patient studies, as reported by Kramer et al. [23]. Their analysis from clinical studies revealed that the kidney-absorbed dose of ^177^Lu-PSMA-617 was ~4-fold lower than the albumin-based agents, ^177^Lu-EB-PSMA-617 and ^177^Lu-PSMA-ALB-56. In addition, the tumor-to-red-marrow values of ^177^Lu-EB-PSMA-617 and ^177^Lu-PSMA-ALB-56 were >10-fold and >5-fold higher than ^177^Lu-PSMA-617, respectively [23]. As a result, the tumor dose at maximum injectable activity was ^177^Lu-EB-PSMA-617 (60.1 Gy), and ^177^Lu-PSMA-ALB-56 (96 Gy) was significantly lower than ^177^Lu-PSMA-671 (131 Gy). Although speculative, projecting mouse data to human data, the estimated tumor radiation doses of ^177^Lu-Alb-L4 (84 Gy) and ^177^Lu-Alb-L6 (94 Gy), respectively, were in the range of the reported agents, considering red marrow as the dose-limiting organ. Following similar dosimetry logistics, the tumor radiation dose of ^177^Lu-L1 (224 Gy) is estimated to be significantly higher than the developed albumin-based agents, considering kidney as the dose-limiting organ. 

Furthermore, we compared these AUC values with our previously reported PSMA-based antibody ^111^In-DOTA-5D3, which was carried out using the same tumor models and same time points [38]. The estimated values are tumor AUC_0-192h_ is 4484 ± 790%ID/g.h, blood AUC_0-192h_ is 2015 ± 251%ID/g.h, and kidney AUC_0-192h_ is 880 ± 38%ID/g.h. The data revealed that the tumor uptake values of the low-molecular-weight albumin-binding agents, such as ^177^Lu-Alb-L4, are significantly (~3-fold) higher than those of the large antibody-based agent, as anticipated. In comparison, the blood AUC of ^177^Lu-Alb-L4 is significantly lower (>6-fold) than ^111^In-DOTA-5D3, albeit with increased kidney uptake (>4-fold). The data suggest that an optimized albumin-binding agent might be a superior option for PSMA-based radiopharmaceutical therapy compared to antibody-based agents.

Although many reported preclinical studies using albumin-binding PSMA-based ^177^Lu-labeled therapeutics were conducted using the PSMA+ PC3 PIP tumor model, these studies mainly used athymic nude mice for tumor implantation. In contrast, our studies were performed using NSG mice because of our institutional availability of this strain. The other notable variables could be related to the high specific activity of the agents used in our studies obtained through HPLC purification and the relatively large tumor used for the biodistribution studies (Supplementary Table S9). We anticipate that blood and normal tissue uptake data could be a rational indicator to compare the performance of the radiotherapeutics, as revealed in a recent report by Tschan et al. [39]. IBU-based ^177^Lu-Alb-L4 displayed a low binding affinity to blood. Our data suggest that an optimized PEG linker, in combination with an IBU-based moiety, may improve the pharmacokinetics of this class of agents. 

There are a few limitations to the study. We used a transfected cell line (PSMA+ PC3 PIP) which may have displayed an unrealistically high PSMA expression and may not have reflected the natural abundance and heterogeneity of PSMA in human cancer. However, the PSMA+ PC3 PIP and PSMA− PC3 flu cells have the advantage of being isogenic and androgen-independent cell lines and are anticipated to display similar biological factors for evaluating tumor pharmacokinetics, except for the PSMA expression levels. Many reported studies currently use the same tumor models for developing similar structure–activity relationship data because of the fast and predictable growth rate. There might be some variability associated with tumor sizes, although the data related to the pharmacokinetic performance are expected to be similar.

## 4. Materials and Methods

General Methods. All reagents and solvents were purchased from Sigma-Aldrich or Fisher Scientific unless specified and are listed in Supplementary Table S1. These were directly used without further purification. Amino acid derivatives were received from Chem-Impex International. 1,4,7,10-Tetraazacyclododecane-1,4,7,10-tetraacetic acid mono-N-hydroxysuccinimide ester (DOTA-NHS-ester) was purchased from Macrocyclics Inc, Dallas. (2S)-2-[[(1S)-1-carboxy-3-methylbutyl]carbamoylamino]pentanedioic acid (ZJ43) was synthesized in-house following that reported in [31]. All new compounds were synthesized using standard solution-phase chemistry based on our well-established methods [27]. Column chromatography of intermediates was performed using Biotage Isolera Flash Chromatography with SNAP Ultra C_18_ Sep-Pak columns. Purification of final compounds was performed using an Agilent (Santa Clara, CA, USA) high-performance liquid chromatography (HPLC) system equipped with a model 1200 quaternary pump and a model 1200 UV absorbance detector using a 250 mm × 10 mm Phenomenex Luna C18 column. Spectral characterization data of the new agents are included in Supplementary Materials. ^177^LuCl_3_ was supplied by the US Department of Energy Isotope Program. All ^177^Lu-labeled radioligands were purified via HPLC to remove unreacted ligand from the radiolabeled material to ensure high specific radioactivity. Animal studies were undertaken according to the guidelines set forth by Johns Hopkins Animal Care and Use Committee. 

### 4.1. Radiochemistry

Radiolabeling was performed under standard labeling conditions in ammonium acetate buffer (0.2 M) at pH ~4.5 following our reported method [27]. The radiolabeling was performed in a radiochemistry microwave chamber at 90 °C for 5 min at 40 watts (Resonance Instruments Inc., Skokie, IL, USA), and the reaction solution was purified using reverse-phase HPLC. An isocratic HPLC method was developed in each case, as listed in Supplementary Table S2, to remove the unreacted ligand from the radiolabeled material to ensure high specific activity. L-Ascorbic acid was added to the isolated radiolabeled compounds in the final formulation to maintain stability and was used for in vitro and in vivo experiments. All ^177^Lu-labeled compounds were stable for up to 4 h at room temperature without significant radiolysis and were stable at 4 °C for 7 d at a concentration of 37 MBq/mL. 

### 4.2. Measurement of Partition Coefficients

The partition coefficient of the ^177^Lu-labeled agents was determined in 1-octanol and phosphate-buffered saline (PBS) (pH 7.4). 1-Octanol (3 mL) and PBS (3 mL) were pipetted into a 15 mL test tube containing 370 kBq of the test compound. The test tube was vortexed for 2 min and then centrifuged (4000× *g*, 5 min). Aliquots (0.1 mL) from the 1-octanol and PBS phases were transferred into two test tubes for counting. The amount of radioactivity in each test tube was measured using the automated γ-counter. The partition coefficient was calculated using the following equation: log P_ow_ = log[counts_1-octanol_/counts_PBS_], and the data are listed in Supplementary Table S3.

### 4.3. In Vitro Assays

#### 4.3.1. Competitive Inhibition Assays 

NAALADase Assay. Binding affinities of all new ligands were measured according to a previously described competitive fluorescence-based assay [40]. In brief, cell lysates of LNCaP cell extracts were incubated with PSMA-targeted agents (0.01 nm–100 μM) in the presence of 4 μM NAAG at 37 °C for 2 h and the reference PSMA inhibitor, ZJ43 (0.01 nm–100 μM) [31]. The amount of released glutamate from NAAG was measured by incubating it with a working solution of the Amplex Red glutamic acid kit (Molecular Probes Inc., Eugene, OR, USA) at 37 °C for 60 min. Fluorescence was measured with excitation at 535 nm and emission at 590 nm using a microplate reader. Inhibition curves were determined using semi-log plots. Data were analyzed using a one-site total binding regression using GraphPad Prism version 9 for Windows (GraphPad Software, San Diego, CA, USA). The IC_50_ values were determined as the concentration at which enzymatic activity was inhibited by 50%. Assays were performed in triplicate, with the entire inhibition study repeated at least once. Enzyme inhibitory constants (*K*_i_ values) were generated using the Cheng–Prusoff conversion [41]. 

#### 4.3.2. Protein-Binding Assay

Albumin-binding properties of the compounds were analyzed following a reported method [42]. A solution of ^177^Lu-Alb-L2–^177^LuAlb-L5 (74 kBq in 10 μL PBS) was added to 190 μL human serum albumin solution (45 mg HSA in 1 mL of PBS). After mixing, the solution was incubated at 37 °C for 60 min. Then, a 100 μL of the reaction solution was loaded onto a gel filtration column (Thermo Scientific™ Zeba™ Spin 7K MWCO size exclusion spin columns, Waltham, MA, USA), previously equilibrated with 0.1 M acetate buffer (pH 6.0), followed by centrifugation (1500× *g*, 2 min). The radioactivity of the column and eluate was then measured using an automated γ-counter. The data are listed in Supplementary Table S3.

#### 4.3.3. Cell Culture

Androgen-independent PSMA-high (PSMA+) PC3 PIP cells and PSMA-low (PSMA−) PC3 flu cell lines are an isogenic subline pair of human PC3 cell lines (androgen-independent PSMA-negative bone metastatic prostate carcinoma). These cell lines were generously provided by Warren Heston (Cleveland Clinic). According to the literature reports, PC3 PIP cells were initially generated via the transfection of PC3 cells, employing VSV-G pseudo-typed lentiviral-vector-expressing human PSMA [43,44,45]. As reported previously, flow cytometry and Western blot assays are routinely used to evaluate PSMA expressions of PSMA+ PC3 PIP cells and PSMA− PC3 flu cells [27]. Selected data generated for the studies of this report are provided in Supplementary Figure S1. These cells were cultured in RPMI-1640 cell culture medium supplemented with 10% fetal calf serum, L-glutamine, antibiotics, and puromycin (2 µg/mL) to maintain expression, and were used for in vitro cell uptake studies and in vivo tumor generation. All cell cultures were maintained at 5% carbon dioxide at 37 °C in a humidified incubator. Authentication of the cell lines were performed routine by JHU GRCF (https://grcf.jhmi.edu/biorepository-cell-center/bioprocessing/cell-line-authentication). We tested mycoplasma contaminations of the cell line cultures every two weeks using the MycoAlert PLUS mycoplasma detection kit (Lonza). 

#### 4.3.4. Cell Uptake and Internalization Study

Cell uptake studies were performed following our previously reported protocol [27]. In brief, adherent PSMA+ PC3 PIP cells and PSMA− PC3 flu cells detached using nonenzymatic buffer (Gibco) and ~1 million cells per tube were incubated in 100 µL of each radiolabeled agent (370 kBq/mL) for 2 h and 24 h at 37 °C in the 100 µL growth medium (binding buffer (1× PBS + 2mM EDTA + 0.5% FBS)). After incubation, the medium was removed at the indicated time points, and the cells were washed three times with ice-cold PBS. The collected pooled washes and the cell pellets were counted using an automated γ-counter. The radioactivity values were converted into a percentage of incubated dose (%ID) per million cells. Experiments were performed in triplicate and repeated two times. 

For the PSMA blocking studies, PSMA+ PC3 PIP cells were pre-incubated with ZJ43 (10 µM final concentration) for 30 min and then washed 3 times with binding buffer followed by incubation of the radioactive dose (100 µL of 370 kBq/mL in binding buffer) for 2 h. The cell uptake studies were then conducted using the method mentioned in the previous section.

For the internalization assays, cells were detached using nonenzymatic buffer, and aliquots of 1 million cells per tube were incubated with 370 kBq/mL of each radiolabeled agent for 2 h and 24 h at 37 °C along with the 100 µL of the binding buffer, as mentioned in the cell uptake study. At the indicated time points, the medium was removed, and cells were washed with binding buffer followed by a mildly acidic buffer (50 mM glycine, 150 mM NaCl (pH 3.0)) at 4 °C for 5 min. The acidic buffer was then collected, and cells were washed twice with binding buffer. The collected pooled washes (containing cell-surface-bound ^177^Lu-Alb-L1–^177^Lu-Alb-L6) and cell pellets (containing internalized ^177^Lu-Alb-L1–^177^Lu-Alb-L6) were counted in an automated γ-spectrometer along with the standards. All radioactivity values were converted into a percentage of incubated dose (%ID) per million cells. Experiments were performed in triplicate and repeated 2 times. Data were fitted according to linear regression analysis.

#### 4.3.5. Clonogenic Survival Assay

Cells (200–1000) were seeded in 60 mm culture dishes. Each radioligand (^177^Lu-L1, ^177^Lu-Alb-L2, ^177^Lu-Alb-L5) was diluted in a prewarmed medium at different concentrations (0, 0.37, 1.85, 3.7, 18.5, 37, 185, and 370 kBq/mL) and incubated with the cells for 48 h, as we previously reported [27]. The radiolabeled compound was replaced with fresh medium, and cells were incubated for 2 weeks or until colonies had at least 50 cells. The colonies were stained with crystal violet and counted, and the surviving fraction was normalized to the control plating efficiency, as previously described [46].

### 4.4. In Vivo Experiments

#### Biodistribution 

Five-to-six-week-old male NSG mice were purchased from Johns Hopkins University research animal resources. Briefly, ~14–20 days after subcutaneous inoculation of PSMA+ PC3 PIP (3 × 10^6^ cells) or PSMA− flu cells (1 × 10^6^ cells) in 100 µL HBSS solution on the upper flanks, tissue biodistribution studies were performed. Male NSG mice bearing PSMA+ PC3 PIP and PSMA− PC3 flu xenografts were injected intravenously with the respective ^177^Lu-labeled agent, ^177^Lu-Alb-L2–^177^Lu-Alb-L6 (1.85 MBq) diluted in 150 µL saline. Mice were sacrificed at 2 h, 24 h, 48 h, and 192 h post-injection, and selected tissues were harvested, weighed, and measured radioactivity using an automated γ-counter. A group of 4 mice was used for each time point; the results were listed as the percentage of the injected dose per gram of tissue mass (%ID/g). The data are presented as the average ± standard deviation (SD). The biodistribution study of ^177^Lu-L1 was performed at only 24 h during the biodistribution study of ^177^Lu-Alb-L2 and ^177^Lu-Alb-L5 in a single experiment. The biodistributions of ^177^Lu-Alb-L3, ^177^Lu-Alb-L4, and ^177^Lu-L1 (24 h) were acquired in a separate experiment. The data are listed in Supplementary Tables S4–S10. Biodistribution data of ^177^Lu-L1 at 3 h, 24 h, 48 h, and 72 h were obtained from our previous report [27]. The 192 h post-injection data of ^177^Lu-L1 are unpublished and were acquired during the same study.

### 4.5. Statistical Analysis

All graphs, AUC calculations, and statistical analyses were created and performed using the GraphPad Prism software (version 9.0). Significant differences were evaluated using a one-way ANOVA or unpaired *t*-test; *p*-values < 0.05 were considered to be significant. *p*-values lower than 0.05 (*p* < 0.05), *p* < 0.01, *p* < 0.001, and *p* < 0.0001 were referred to with one (*), two (**), three (***), or four (****) asterisks, respectively.

## 5. Conclusions

The data suggest that ^177^Lu-Alb-L4 could be an improved option for ^177^Lu-L1, and further modification in the linker construct using a PEG linker may be possible to reduce blood and kidney uptake. 

## Data Availability

Not applicable.

## Supplementary Materials

The following supporting information can be downloaded at: https://www.mdpi.com/article/10.3390/molecules28166158/s1, Figure S1: Validation of PSMA expression in isogenic human prostate cancer PC3 sublines, PSMA+ PC3 PIP and PSMA− PC3 flu. PSMA+ PC3 PIP is designed to overexpress copious amounts of PSMA. A. PSMA total protein levels in PSMA+ PC3 PIP and PSMA− PC3 flu cells by western blot analysis. B. Real time PCR PSMA gene expression in PSMA+ PC3 PIP and PSMA− PC3 flu cells. C. Flow cytometry data showing PSMA surface expression on PSMA+ PC3 PIP and PSMA− flu cells; Table S1: List of chemicals and solvents used in this report; Table S2. HPLC Methods for 177Lu-Alb-L1-177Lu-Alb-L6; Table S3. Partition coefficient (POctanol/water) and albumin binding properties of ^177^Lu-Alb-L2-^177^Lu-Alb-L6; Table S4. Tissue biodistribution data of ^177^Lu-Alb-L2 in male NSG mice bearing PSMA+ PC3 PIP and PSMA− PC3 flu xenografts in either flank (Data presented in % ID/g, expressed as mean ± SD) (n = 4). Table S5. Tissue biodistribution data of 177Lu-Alb-L3 in male NSG mice bearing PSMA+ PC3 PIP and PSMA− PC3 flu xenografts in either flank (Data presented in % ID/g, expressed as mean ± SD) (n = 4). Table S6. Tissue biodistribution data of 177Lu-Alb-L4 in male NSG mice bearing PSMA+ PC3 PIP and PSMA− PC3 flu xenografts in either flank (Data presented in % ID/g, expressed as mean ± SD) (n = 4). Table S7. Tissue biodistribution data of 177Lu-Alb-L5 in male NSG mice bearing PSMA+ PC3 PIP and PSMA− PC3 flu xenografts in either flank (Data presented in % ID/g, expressed as mean ± SD) (n = 4). Table S8. Tissue biodistribution data of 177Lu-Alb-L6 in male NSG mice bearing PSMA+ PC3 PIP xenografts (Data presented in %ID/g, expressed as mean ± SD) (n = 4). Table S9. PSMA+ PC PIP tumor weight (g) and expressed as mean ± SD) and tumor volume (mm^3^, expressed as mean ±SD) used for biodistribution studies. Table S10. Tissue biodistribution data of 177Lu-Alb-L1 in male NSG mice bearing PSMA+ PC3 PIP and PSMA− PC3 flu xenografts in either flank (Data presented in % ID/g, expressed as mean ± SD) (n = 3–4) at 24 h post-injection. The biodistribution studies of 177Lu-Alb-L2, 177Lu-Alb-L5, and 177Lu-L1(A, 24 h) were done using the same batch of mice and the same radioactivity. The biodistribution studies of 177Lu-Alb-L3, 177Lu-Alb-L4, and 177Lu-L1 (B, 24 h) were done head-to-head using the same batch of mice and radioactivity. The data are presented below.
