# Supplementary material for: Preclinical Evaluation of a New Series of Albumin-Binding 177Lu-Labeled PSMA-Based Low-Molecular-Weight Radiotherapeutics
Source: Molecules. 2023 Aug 21;28(16):6158. doi: 10.3390/molecules28166158 (PMC10459686; doi:10.3390/molecules28166158)
Supplement: Supplementary file 1 [file molecules-28-06158-s001.zip › molecules-2484071-supplementary.pdf]

Supplementary Material

Preclinical evaluation of a new series of albumin-binding ^{177}Lu-labeled PSMA-based low-molecular-weight radiotherapeutics

Srikanth Boinapally[1], Suresh Alati,[1] Zirui Jiang[1], Rajan Singh[1], Yu Yan[1], Alla Lisok[1], Gabriela Lofland, IL Minn[1], Robert F. Hobbs, Martin G. Pomper[1,2], Sangeeta Ray Banerjee[1,2]

[1]Russell H. Morgan Department of Radiology and Radiological Science, [2]Sidney Kimmel Comprehensive Cancer Center, Johns Hopkins University Baltimore, MD, USA

Address correspondence to: Sangeeta Ray Banerjee (sray9@jhmi.edu)

1.	Synthesis, characterization, and Spectral data ESI-HRMS, NMR, and HPLC chromatograms	2-22
2.	Supplementary Figure S1 and biological methods	23-24
3.	Supplementary Tables S1-S10	25-33
4.	Reference	34

Synthesis and characterization of new ligands

New agents were prepared following several solution phase reaction schemes described below.

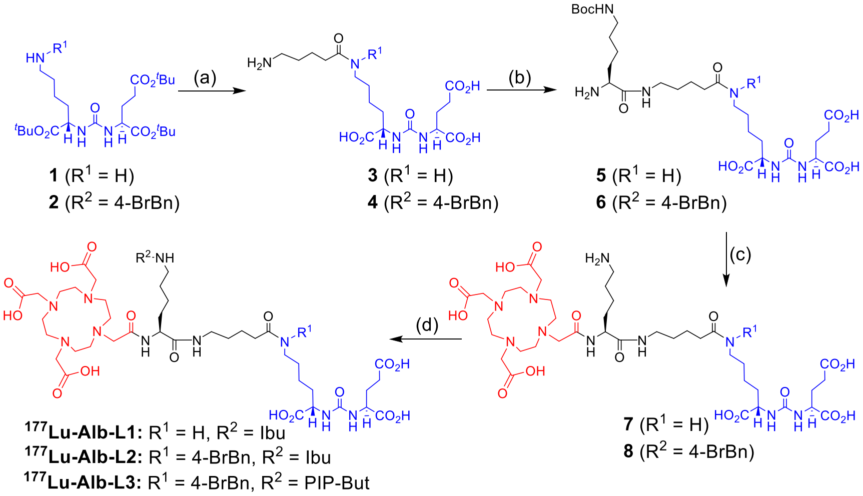

Scheme 1. a) (i) 2,5-dioxopyrrolidin-1-yl 5-((tert-butoxycarbonyl)amino)pentanoate, DIPEA, DMF, RT, overnight. (ii) TFA:CH_2Cl_2 (1:1), RT, 2 h; b) (i) Fmoc-L-Lys(Boc)-OSu, DIPEA, DMF, RT, 2 h (ii) 20% piperidine, DMF, RT, 1 h; c) (i) DOTA-NHS-ester, DIPEA, DMSO, RT, 2 h. (ii) TFA:CH_2Cl_2 (1:1), RT, 2 h; d) IBU-NHS or IPBA-NHS, DIPEA, DMSO, RT, 2 h.

Scheme S1. Protected Glu-Lys-urea (KEU) 1 or 2 was prepared from commercially available starting materials and following our previously reported methods (1). Initially, KEU was treated

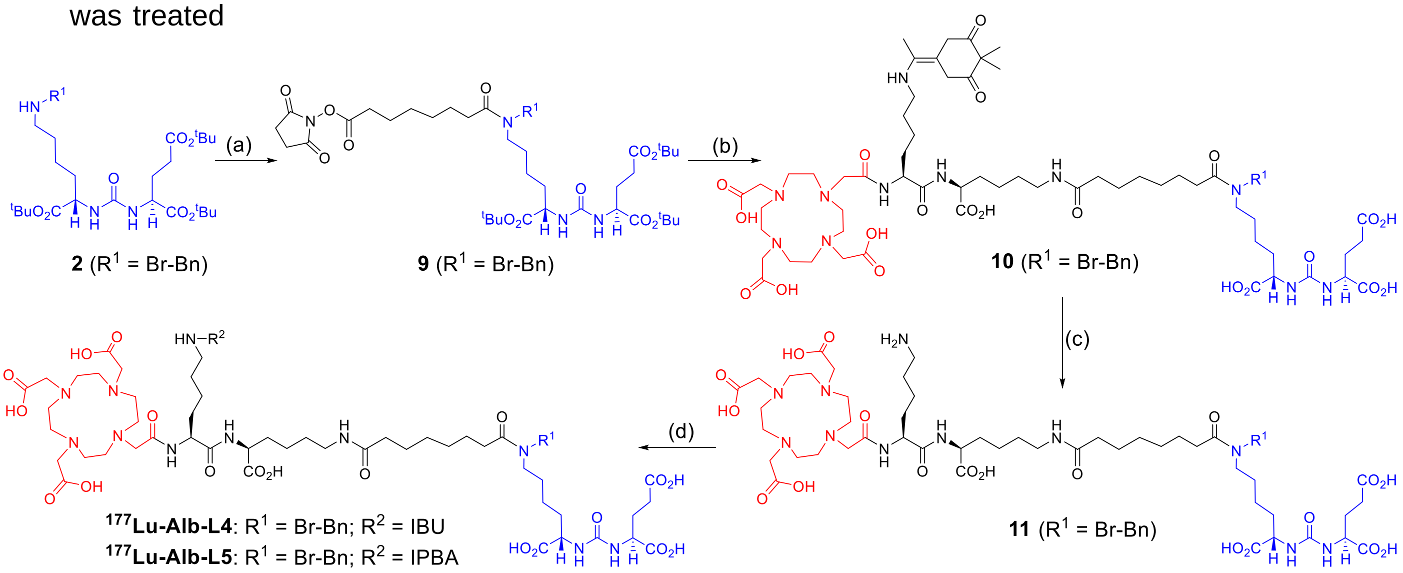

Scheme S2. a) DSS, DIPEA, DMF, RT, overnight; b) (i) DOTA-L-Lys(Dde)-L-Lys-NH_2, DIPEA, DMSO, RT, 2 h, 65% (ii) TFA:CH_2Cl_2 (1:1), RT, 2 h, 70%; c). 2% hydrazine-hydrate in DMF, RT, 30 min, 66%; d) IBU-NHS or IPBA-NHS, DIPEA, DMSO, RT, 2 h, 88%.

with 2,5-dioxopyrrolidin-1-yl 5-((tert-butoxycarbonyl)amino)pentanoate in the presence of DIPEA, followed by deprotection using trifluoroacetic acid (TFA) and methylene dichloride (DCM), which provides the corresponding intermediate compound 3 or 4. Then adding Fmoc-L-Lys(Boc)-OSu to the intermediate compound 3 or 4; removing Boc using TFA provided the intermediate compound 5 or 6. Next, chelating agent DOTA-NHS-ester was added to the intermediate 5 or 6 to create compound 7 or 8. Subsequently, the Boc group of lysine was removed from compounds 7 or 8. The crude product was conjugated with ibuprofen (IBU) or 4-(*p*-iodophenyl) butyric acid (IPBA) to create Alb-L1, Alb-L1, and Alb-L3.

In **Scheme S2**, the protected Glu-Lys-urea (KEU), 2, was treated with disuccinimidyl suberate (DSS) following our reported method to create the intermediate compound 9 in good yield (1). Then, compound 9 was treated with DOTA-L-Lys(Dde)-L-Lys-NH_2 in the presence of DIPEA, followed by deprotection, which produced the intermediate 10 in good yield (65%). Subsequently, compound 10 was converted into the corresponding amine derivatives 11 with 2% hydrazine hydrate for 2 min. Next, the resultant crude compound was treated with IBU-NHS or IPBA -But-NHS, which delivered ^{177}Lu-Alb-L4 or ^{177}Lu-Alb-L5 in good yield.

In **Scheme S3,** for creating ^{177}Lu-Alb-L6, compound 2 was reacted with 2,5-dioxopyrrolidin-1-yl 6-((tert-butoxycarbonyl)amino)hexanoate to generate the corresponding intermediate 12, which was then treated with t-Boc-N-amido-PEG10-NHS ester, to deliver compound 13. Next, Fmoc-L-Lys(Boc)-OSu was added to compound 13, followed by deprotection and addition of DOTA-NHS-ester generated compound 14. Finally, 14 was conjugated with IPBA-NHS to provide ^{177}Lu-Alb-L6 in > 50% yields.

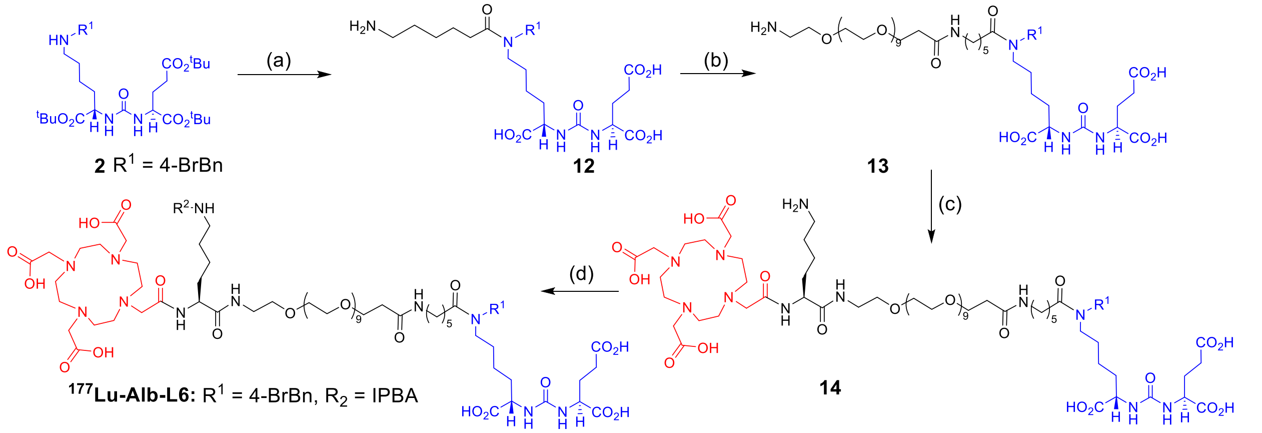

Scheme S 3. a) (i) 2,5-dioxopyrrolidin-1-yl 6-((*tert*-butoxycarbonyl)amino)hexanoate, DIPEA, DMF, RT, overnight. (ii) TFA:CH_2Cl_2 (1:1), RT, 2 h; b) (i) t-Boc-N-amido-PEG10-NHS ester, DMSO, TEA, RT, 2 h. (ii) TFA:CH_2Cl_2 (1:1), RT, 2 h; c) (i) Fmoc-L-Lys(Boc)-OSu, TEA, DMSO, RT, 2 h. (ii) 20% piperidine, DMF, RT, 1 h. iii) DOTA-NHS-ester, DIPEA, DMSO, RT, 2 h. (iv) TFA:CH_2Cl_2 (1:1), RT, 2 h; d) IPBA-NHS, DIPEA, DMSO, RT, 2 h.

(((*S*)-5-(5-Aminopentanamido)-1-carboxypentyl)carbamoyl)-L-glutamic acid (3): To a stirred solution of **1** (200 mg, 0.410 mmol, 1.0 eq) and 2,5-dioxopyrrolidin-1-yl 5-((tert-butoxycarbonyl)amino)pentanoate (129 mg, 0.410 mmol, 1.0 eq) in DMF (2 mL) was added diisopropyl ethyl amine (DIPEA) (214 µL, 1.23 mmol, 3.0 eq) at room temperature. The reaction mixture was stirred overnight and concentrated to isolate the crude product. To the above crude was added 2 mL of TFA/CH_2Cl_2 (1:1) at room temperature, and the mixture was stirred for 2 h. The reaction mixture was then concentrated and purified using acetonitrile (ACN)/water (H_2O) on the C18 Sep-Pak column to provide compound **3** (146 mg, 85%) as a white solid. ^{1}H NMR (500 MHz, D_2O) δ 4.25 (dd, *J* = 5.5, 9.5 Hz, 1H), 4.17 (dd, *J* = 5.0, 9.0 Hz, 1H), 3.17 (t, *J* = 6.5 Hz, 2H), 3.02-2.92 (m, 2H), 2.50 (t, *J* = 7.0 Hz, 2H), 2.30-2.22 (m, 2H), 2.21-2.11 (m, 1H), 2.01-1.90 (m, 1H), 1.88-1.76 (m, 1H), 1.74-1.58 (m, 5H), 1.55-1.45 (m, 2H), 1.44-1.30 (m, 2H).

(10*S*,23*S*,27*S*)-10-Amino-2,2-dimethyl-4,11,17,25-tetraoxo-3-oxa-5,12,18,24,26-pentaaza nonacosane-23,27,29-tricarboxylic acid (5): To a stirred solution of **3** (60 mg, 0.143 mmol, 1.0 eq) and Fmoc-L-Lys(Boc)-OSu (81 mg, 0.143 mmol, 1.0 eq) in DMF (1 mL) was added DIPEA (124 µL, 0.717 mmol, 5.0 eq)) at room temperature. The reaction mixture was stirred for 2 h and concentrated to get crude product. To the above crude was added 2 mL of 20% piperidine in DMF

(2 mL) at room temperature, and the mixture was stirred for 1 h. Concentrated and purified using ACN/H_2O on the C18 Sep-Pak column to provide compound **5** (65 mg, 70%) as a white solid. HRMS (ESI) m/z: $[M + H]^+$ calcd for $C_{28}H_{51}N_6O_{11}$, 647.3611; found, 647.3610.

(3*S*,7*S*,20*S*)-27-(4-Isobutylphenyl)-5,13,19,26-tetraoxo-20-(2-(4,7,10-tris(carboxymethyl)-1,4,7,10-tetraazacyclododecan-1-yl)acetamido)-4,6,12,18,25-pentaazaoctacosane-1,3,7-tricarboxylic acid (^{177}Lu-Alb-L1): To a stirred solution of **5** (22.5 mg, 0.034 mmol, 1.0 eq) and DOTA-NHS-ester (29.13 mg, 0.038 mmol, 1.1 eq) in DMSO (500 µL) was added DIPEA (72 µL, 0.417 mmol, 12.0 eq) at room temperature. The reaction mixture was then stirred for 2 h at room temperature and purified using ACN/H_2O on the C18 Sep-Pak column to provide the desired compound as a white solid. To the isolated compound (26.8 mg, 0.026 mmol), was added 2 mL of TFA/CH_2Cl_2 (1:1) at room temperature. The reaction mixture was then stirred for 2 h and concentrated to produce compound 7. Compound 7 was used for the next step without further purification. To the stirred solution of 7 and 2,5-dioxopyrrolidin-1-yl-2-(4-isobutyl phenyl)propanoate (8.09 mg, 0.026 mmol) in DMSO (500 µL) was added DIPEA (46 µL, 0.266 mmol, 12.0 eq) at room temperature. The reaction mixture was stirred for 2 h, and the crude product obtained after solvent evaporation was purified by preparative RP-HPLC chromatography using 0.1% TFA in H_2O and 0.1% TFA in acetonitrile as eluents followed by lyophilization afforded compound **^{177}Lu-Alb-L1** (23.4 mg, 60%) as a white solid. [RP-HPLC purification was achieved using Agilent System, λ 220 nm, 250 mm x 10 mm Phenomenex Luna C_{18} column, solvent gradient: 90% H_2O (0.1% TFA) and 10% ACN (0.1% TFA), reaching 60% of ACN in 20 min at a flow rate of 10 mL/min, product eluted at 11.3 min]. ^1H NMR (500 MHz, DMSO-d_6) δ 8.64 (s, 1H), 8.03 (s, 1H), 7.93 (s, 1H), 7.79 (s, 1H), 7.18 (d, *J* = 7.5 Hz, 2H), 7.06 (d, *J* = 7.5 Hz, 2H), 6.38-6.25 (m, 2H), 4.22-4.14 (m, 2H), 4.12-4.05 (m, 2H), 3.07 (s, 8H), 3.01-2.86 (m, 6H), 2.50 (s, 16H), 2.38 (d, *J* = 7.0 Hz, 2H), 2.36-2.16 (m, 4H), 2.03 (t, *J* = 6.5 Hz, 2H), 1.97-1.86 (m, 1H), 1.82-1.10

(m, 16H), 1.28 (d, *J* = 7.0 Hz, 3H), 0.84 (d, *J* = 6.5 Hz, 6H); HRMS (ESI) m/z: $[M + H]^+$ calcd for $C_{52}H_{85}N_{10}O_{17}$, 1121.6100; found, 1121.6088.

(((*S*)-5-(5-Amino-N-(4-bromobenzyl)pentanamido)-1-carboxypentyl)carbamoyl)-L-glutamic acid (4): To a stirred solution of 2 (214 mg, 0.326 mmol, 1.0 eq) and 2,5-dioxopyrrolidin-1-yl 5-((tert-butoxycarbonyl)amino)pentanoate (102.5 mg, 0.326 mmol, 1.0 eq) in DMF (2 mL) was added DIPEA (170 µL, 0.978 mmol, 3.0 eq) at room temperature. The reaction mixture was stirred for 24 h and concentrated to get crude product. To the above crude was added 2 mL of TFA/CH_2Cl_2 (1:1) at room temperature, and the reaction mixture was stirred for 2 h. The reaction mixture was then concentrated and purified using ACN/H_2O on the C18 Sep-Pak column to provide compound 4 (143 mg, 75%) as a white solid. ^1H NMR (500 MHz, D_2O) δ 7.55 (d, *J* = 8.5 Hz, 1H), 7.51 (d, *J* = 8.5 Hz, 1H), 7.12 (dd, *J* = 2.5, 8.0 Hz, 2H), 4.59 (s, 1H), 4.51 (s, 1H), 4.29-4.22 (m, 1H), 4.19-4.11 (m, 1H), 3.39-3.29 (m, 2H), 3.05-2.88 (m, 2H), 2.59-2.38 (m, 4H), 2.22-2.10 (m, 1H), 2.02-1.90 (m, 1H), 1.82-1.46 (m, 8H), 1.39-1.24 (m, 2H).

(10*S*,23*S*,27*S*)-10-Amino-18-(4-bromobenzyl)-2,2-dimethyl-4,11,17,25-tetraoxo-3-oxa-5,12,18,24,26-pentaazanonacosane-23,27,29-tricarboxylic acid (6): To a stirred solution of 4 (139 mg, 0.236 mmol, 1.0 eq) and Fmoc-L-Lys(Boc)-OSu (133.9 mg, 0.236 mmol, 1.0 eq) in DMF (2 mL) was added DIPEA (250 µL, 1.42 mmol, 6.0 eq) at room temperature. The reaction mixture was stirred for 2 h and concentrated to get the crude product. To the isolated crude was added 2 mL of 20% piperidine in DMF (3 mL) at room temperature, and the mixture was stirred for 1 h. Concentrated and purified using ACN/H_2O on the C18 Sep-Pak column to provide compound 6 (125 mg, 65%) as a white solid. ^1H NMR (500 MHz, DMSO-d_6) δ 8.43-8.32 (m, 1H), 8.08 (s, 2H), 7.93 (d, *J* = 7.5 Hz, 1H), 7.74 (d, *J* = 7.5 Hz, 1H), 7.55 (d, *J* = 8.0 Hz, 1H), 7.41 (d, *J* = 8.0 Hz, 1H), 6.74 (t, *J* = 5.0 Hz, 1H), 6.40-6.26 (m, 2H), 4.52 (s, 1H), 4.44 (s, 1H), 4.14-3.98 (m, 2H), 3.23-2.97 (m, 5H), 2.93-2.82 (m, 2H), 2.37 (t, *J* = 7.0 Hz, 1H), 2.31-2.15 (m, 3H), 1.96-1.43 (m,

14H), 1.35 (s, 9H), 1.29-1.15 (m, 4H); $[M + H]^+$ calcd for $C_{35}H_{56}BrN_6O_{11}$, 815.3192; found, 815.3184.

(3*S*,7*S*,20*S*)-12-(4-Bromobenzyl)-27-(4-isobutylphenyl)-5,13,19,26-tetraoxo-20-(2-(4,7,10-tris(carboxymethyl)-1,4,7,10-tetraazacyclododecan-1-yl)acetamido)-4,6,12,18,25-pentaazaoctacosane-1,3,7-tricarboxylic acid (^{177}Lu-Alb-L2): To a stirred solution of 6 (33.3 mg, 0.04 mmol, 1.0 eq) and DOTA-NHS-ester (34.2 mg, 0.045 mmol, 1.1 eq) in DMSO (500 µL) was added DIPEA (85 µL, 0.490 mmol, 12.0 eq) at room temperature. The reaction mixture was stirred for 2 h and purified using acetonitrile/H_2O on the C18 Sep-Pak column to provide the desired product as a white solid (41 mg, 85% yield). To the crude product (20 mg, 0.016 mmol) was added 2 mL of TFA/CH_2Cl_2 (1:1) at room temperature; the reaction mixture was stirred for 2 h and concentrated to afford a crude compound 8. To the stirred solution of crude 8 (18.3 mg, 0.0166 mmol, 1.0 eq) and 2,5-dioxopyrrolidin-1-yl 2-(4-isobutylphenyl)propanoate (6.0 mg, 0.0199 mmol, 1.2 eq) in DMSO (500 µL) was added DIPEA (35 µL, 0.199 mmol, 12.0 eq)) at room temperature. The reaction mixture was stirred for 2 h, and the resultant reaction mixture was purified by preparative RP-HPLC chromatography using 0.1% TFA in H_2O and 0.1% TFA in acetonitrile as eluents followed by lyophilization afforded **^{177}Lu-Alb-L2** (19.3 mg, 90%) as a white solid. [RP-HPLC purification was performed using Agilent System, λ 220 nm, 250 mm × 10 mm Phenomenex Luna C_{18} column, solvent gradient: 90% H_2O (0.1% TFA) and 10% ACN (0.1% TFA), reaching 60% of ACN in 20 min at a flow rate of 10 mL/min, product eluted at 14.6 min]. ^{1}H NMR (500 MHz, DMSO-d_6) δ 8.63 (s, 1H), 8.03 (d, *J* = 8.5 Hz, 1H), 7.91 (s, 1H), 7.54 (d, *J* = 7.5 Hz, 1H), 7.48 (d, *J* = 8.0 Hz, 1H), 7.23-7.10 (m, 4H), 7.05 (d, *J* = 7.0 Hz, 2H), 6.39-6.25 (m, 2H), 4.50 (s, 1H), 4.43 (s, 1H), 4.26-4.00 (m, 4H), 3.07 (s, 8H), 2.96-2.73 (m, 6H), 2.50 (s, 16H), 2.37 (d, *J* = 7.5 Hz, 2H), 2.30-2.15 (m, 4H), 1.96-1.85 (m, 1H), 1.82-1.56 (m, 4H), 1.55-1.11 (m, 14H), 1.27 (d, *J* = 7.0 Hz, 3H), 0.83 (d, *J* = 6.0 Hz, 6H); HRMS (ESI) m/z: $[M + H]^+$ calcd for $C_{59}H_{90}BrN_{10}O_{17}$, 1289.5642; found, 1289.5663.

(3*S*,7*S*,20*S*)-12-(4-bromobenzyl)-29-(4-iodophenyl)-5,13,19,26-tetraoxo-20-(2-(4,7,10-tris(carboxymethyl)-1,4,7,10-tetraazacyclododecan-1-yl)acetamido)-4,6,12,18,25-pentaazanonacosane-1,3,7-tricarboxylic acid (^{177}Lu-Alb-L3): To a stirred solution of 6 (35.2 mg, 0.043 mmol, 1.0 eq) and DOTA-NHS-ester (34.5 mg, 0.045 mmol, 1.05 eq) in DMSO (300 µL) was added DIPEA (90 µL, 0.518 mmol, 12.0 eq) at room temperature. The reaction mixture was stirred for 2 h and purified using ACN/H_2O on the C18 Sep-Pak column to provide the desired product as a white solid (41 mg, 80% yield). To the above product (29 mg, 0.016 mmol) was added 2 mL of TFA/CH_2Cl_2 (1:1) at room temperature; the reaction mixture was stirred for 2 h and concentrated to afford a crude amine 8. To the stirred solution of crude 8 (26.5 mg, 0.0240 mmol, 1.0 eq) and 2,5-dioxopyrrolidin-1-yl 4-(4-iodophenyl)butanoate (9.77 mg, 0.0252 mmol, 1.05 eq) in DMSO (300 µL) was added DIPEA (50 µL, 0.288 mmol, 12.0 eq) at room temperature. The reaction mixture was stirred for 2 h, and crude was purified by reverse phase flash chromatography using 0.1% TFA in H_2O and 0.1% TFA in acetonitrile as eluents followed by lyophilization afforded compound **^{177}Lu-Alb-L3** (19.8 mg, 60%) as a white solid. [Flash chromatography purification was achieved using Biotage Isolera One system, λ 220 nm, Biotage SNAP Ultra C18 column (12 g), solvent gradient: 90% H_2O (0.1% TFA) and 10% ACN (0.1% TFA), reaching 90% of ACN, product eluted at 50% to 55% of (B) in (A) fraction]. ^{1}H NMR (500 MHz, DMSO-d_6) δ 8.89-8.58 (m, 1H), 8.41 (s, 1H), 7.96-7.89 (m, 1H), 7.60 (d, J = 8.0 Hz, 2H), 7.53 (d, J = 8.0 Hz, 1H), 7.48 (d, J = 8.5 Hz, 1H), 7.14 (d, J = 7.0 Hz, 2H), 7.00 (d, J = 8.0 Hz, 2H), 6.71-6.35 (m, 2H), 4.43 (s, 2H), 4.20-4.09 (m, 1H), 4.04-3.95 (m, 2H), 3.51-2.86 (m, 14H), 2.50 (s, 16H), 2.39-2.31 (m, 2H), 2.27-2.18 (m, 3H), 2.04 (t, J = 7.5 Hz, 2H), 1.80-1.69 (m, 4H), 1.68-1.30 (m, 12H), 1.29-1.13 (m, 5H); HRMS (ESI) m/z: $[M + K]^+$ calcd for $C_{56}H_{82}BrIKN_{10}O_{17}$, 1411.3688; found, 1411.3719.

di-*tert*-Butyl(((*S*)-6-(N-(4-bromobenzyl)-8-((2,5-dioxopyrrolidin-1-yl)oxy)-8-oxooctanamido)-1-(tert-butoxy)-1-oxohexan-2-yl)carbamoyl)-L-glutamate (9): To a solution of DSS (308 mg,

0.838 mmol, 2.2 eq) in 6 mL DMF was added a solution of di-tert-butyl (((S)-6-((4-bromobenzyl)amino)-1-(tert-butoxy)-1-oxohexan-2-yl)carbamoyl dropwise)-L-glutamate **2** (250 mg, 0.381 mmol, 1.0 eq) in 2 mL DMF and TEA (58 µL, 0.419 mmol, 1.1 eq) at rt for 20 min. The reaction mixture was left stirring overnight at room temperature. The solvent was removed under vacuum, and the solid residue was purified using a silica gel column using CH_3CN/CH_2Cl_2 as a solvent system to obtain compound **9** (225 mg, 65%). ^{1}H NMR (500 MHz, $CDCl_3$) δ 7.46 (d, J = 8.0 Hz, 1H), 7.40 (d, *J* = 8.0 Hz, 1H), 7.08 (d, *J* = 8.0 Hz, 1H), 7.02 (d, *J* = 8.0 Hz, 1H), 5.72-5.05 (m, 2H), 4.50 (s, 1H), 4.46 (s, 1H), 4.35-4.20 (m, 2H), 3.39-3.22 (m, 1H), 3.13 (t, J = 7.5 Hz, 1H), 2.80 (s, 4H), 2.58 (dt, *J* = 7.5, 15.0 Hz, 2H), 2.42-2.19 (m, 4H), 2.10-1.98 (m, 1H), 1.89-1.62 (m, 6H), 1.60-1.48 (m, 2H), 1.49-1.34 (m, 30H), 1.33-1.18 (m, 4H); HRMS (ESI) m/z: $[M + H]^+$ calcd for $C_{43}H_{66}BrN_4O_{12}$, 909.3846; found, 909.3855.

(8*S*,11*S*,30*S*,34*S*)-25-(4-Bromobenzyl)-2-(4,4-dimethyl-3,5-dioxocyclohexylidene)-9,17,24,32-tetraoxo-8-(2-(4,7,10-tris(carboxymethyl)-1,4,7,10-tetraazacyclododecan-1-yl)acetamido)-3,10,16,25,31,33-hexaazahexatriacontane-11,30,34,36-tetracarboxylic acid (10): To a stirred solution of 9 (32.5 mg, 0.035 mmol, 1.0 eq) and DOTA-L-Lys(Dde)-L-Lys-NH_2 (29.5 mg, 0.035 mmol, 1.0 eq) in DMSO (300 µL) was added DIPEA (38 µL, 0.214 mmol, 6.0 eq)) at room temperature. The reaction mixture was stirred overnight and concentrated to obtain the crude product. To the above crude was added 2 mL of TFA/CH_2Cl_2 (1:1) at room temperature, and the mixture was stirred for 2 h. Concentrated and purified using ACN/H_2O on C_{18} Sep-Pak column to provide compound 10 (36 mg, 70%) as a white solid. ^{1}H NMR (500 MHz, DMSO-d_6) δ 13.22 (t, *J* = 5.0 Hz, 1H), 8.70 (s, 1H), 8.35 (d, *J* = 5.0 Hz, 1H), 7.83-7.73 (m, 1H), 7.55 (d, *J* = 8.5 Hz, 1H), 7.49 (d, *J* = 8.5 Hz, 1H), 7.17-7.10 (m, 2H), 6.37-6.26 (m, 2H), 4.51 (s, 1H), 4.43 (s, 1H), 4.41-4.33 (m, 1H), 4.18-4.39 (m, 3H), 3.08 (s, 8H), 3.02-2.92 (m, 4H), 2.50 (s, 16H), 2.46 (s, 4H), 2.37-2.30 (m, 2H), 2.26 (s, 3H), 2.27-2.17 (m, 3H), 2.05-1.85 (m, 3H), 1.79-1.08 (m, 28H), 0.93 (s, 6H); HRMS (ESI) m/z: $[M + H]^+$ calcd for $C_{65}H_{101}BrN_{11}O_{21}$, 1450.6362; found, 1450.6351.

(4*S*,7*S*,26*S*,30*S*)-4-(4-Aminobutyl)-21-(4-bromobenzyl)-2,5,13,20,28-pentaoxo-1-(4,7,10-tris(carboxymethyl)-1,4,7,10-tetraazacyclododecan-1-yl)-3,6,12,21,27,29-hexaazadotriacontane-7,26,30,32-tetracarboxylic acid (11): Compound 10 (20 mg, 0.013 mmol) was dissolved 2 mL 2% hydrazine-hydrate in DMF at room temperature followed by 200 µL water. The solution was left stirring at room temperature for 30 min, then evaporated to dryness. The colorless residue was purified using ACN/H_2O on the C18 Sep-Pak column to provide compound 11 (11.6 mg, 66%) as a white solid. ^{1}H NMR (500 MHz, DMSO-d_6) δ 8.61 (s, 1H), 8.28 (s, 1H), 7.81-7.62 (m, 3H), 7.55 (d, *J* = 8.0 Hz, 1H), 7.49 (d, *J* = 8.0 Hz, 1H), 7.17-7.09 (m, 2H), 6.37-6.25 (m, 2H), 4.51 (s, 1H), 4.43 (s, 1H), 4.36-4.28 (m, 1H), 4.17-4.04 (m, 3H), 3.07 (s, 8H), 2.99-2.92 (m, 2H), 2.80-2.67 (m, 4H), 2.50 (s, 16H), 2.38-2.15 (m, 5H), 2.07-1.85 (m, 4H), 1.75-1.10 (m, 25H); HRMS (ESI) m/z: $[M + H]^+$ calcd for $C_{55}H_{89}BrN_{11}O_{19}$, 1286.5504; found, 1286.5514.

(3*S*,7*S*,26*S*,29*S*,36*S*)-12-(4-Bromobenzyl)-36-(4-isobutylphenyl)-5,13,20,28,35-pentaoxo-29-(2-(4,7,10-tris(carboxymethyl)-1,4,7,10-tetraazacyclododecan-1-yl)acetamido)-4,6,12,21,27,34-hexaazaheptatriacontane-1,3,7,26-tetracarboxylic acid (^{177}Lu-Alb-L4): To the stirred solution of amine **11** (7.35 mg, 0.0057 mmol, 1.0 eq) and 2,5-dioxopyrrolidin-1-yl 2-(4-isobutylphenyl)propanoate (2.08 mg, 0.0068 mmol, 1.2 eq) in DMSO (100 µL) was added DIPEA (9.75 µL, 0.057 mmol, 10.0 eq) at room temperature. The reaction mixture was stirred for 2 h, concentrated, and the solid crude was purified by preparative RP-HPLC chromatography using 0.1% TFA in H_2O and 0.1% TFA in acetonitrile as eluents followed by lyophilization afforded compound **^{177}Lu-Alb-L4** (7.4 mg, 88%) as a white solid. [RP-HPLC purification was achieved using Agilent System, λ 220 nm, 250 mm × 10 mm Phenomenex Luna C18 column, solvent gradient: 95% H_2O (0.1% TFA) and 5% ACN (0.1% TFA), reaching 60% of ACN in 20 min at a flow rate of 5 mL/min, product eluted at 18.3 min]. ^{1}H NMR (500 MHz, DMSO-d_6) δ 12.62 (broad singlet, 5H), 8.66 (s, 1H), 8.28 (d, *J* = 7.0 Hz, 1H), 7.92-7.85 (m, 1H), 7.80-7.71 (m, 1H), 7.55 (d,

J = 7.5 Hz, 1H), 7.49 (d, *J* = 7.5 Hz, 1H), 7.22-7.11 (m, 4H), 7.06 (d, *J* = 8.0 Hz, 2H), 6.38-6.25 (m, 2H), 4.51 (s, 1H), 4.44 (s, 1H), 4.36-4.29 (m, 4H), 3.51 (q, *J* = 7.0 Hz, 1H), 3.24-2.84 (m, 14H), 2.50 (s, 16H), 2.38 (d, *J* = 7.0 Hz, 2H), 2.35-2.17 (m, 4H), 2.07-1.85 (m, 3H), 1.83-1.57 (m, 6H), 1.55-1.32 (m, 12H), 1.29-1.11 (m, 13H), 0.84 (d, *J* = 6.5 Hz, 6H); HRMS (ESI) m/z: $[M + H]^+$ calcd for $C_{68}H_{105}BrN_{11}O_{20}$, 1474.6715; found, 1474.6715.

(3*S*,7*S*,26*S*,29*S*)-12-(4-Bromobenzyl)-38-(4-iodophenyl)-5,13,20,28,35-pentaoxo-29-(2-(4,7,10-tris(carboxymethyl)-1,4,7,10-tetraazacyclododecan-1-yl)acetamido)-4,6,12,21,27,34-hexaazaoctatriacontane-1,3,7,26-tetracarboxylic acid (^{177}Lu-Alb-L5): To the stirred solution of amine **11** (8.5 mg, 0.0066 mmol, 1.0 eq) and 2,5-dioxopyrrolidin-1-yl 4-(4-iodophenyl)butanoate (3.0 mg, 0.0079 mmol, 1.2 eq) in DMSO (100 µL) was added DIPEA (11.4 µL, 0.066 mmol, 10.0 eq) at room temperature. The reaction mixture was stirred for 2 h, and the crude was purified by preparative RP-HPLC chromatography using 0.1% TFA in H_2O and 0.1% TFA in acetonitrile as eluents followed by lyophilization afforded compound **^{177}Lu-Alb-L5** (9.0 mg, 88%) as a white solid. RP-HPLC purification was achieved using Agilent System, λ 220 nm, 250 mm × 10 mm Phenomenex Luna C18 column, solvent gradient: 90% H_2O (0.1% TFA) and 10% ACN (0.1% TFA), reaching 60% of ACN in 20 min at a flow rate of 10 mL/min, product eluted at 14.2 min]. ^{1}H NMR (500 MHz, DMSO-d_6) δ 8.37 (s, 1H), 8.25 (s, 1H), 7.85-7.72 (m, 2H), 7.64-7.59 (m, 2H), 7.55 (d, *J* = 8.0 Hz, 1H), 7.49 (d, *J* = 8.0 Hz, 1H), 7.18-7.11 (m, 2H), 7.00 (d, *J* = 8.0 Hz, 2H), 6.38-6.26 (m, 2H), 4.50 (s, 1H), 4.43 (s, 1H), 4.35-4.26 (m, 1H), 4.13-4.04 (m, 2H), 2.99 (s, 8H), 3.05-2.85 (m, 6H), 2.50 (s, 16H), 2.38-2.16 (m, 4H), 2.08-1.88 (m, 3H), 1.78-1.11 (m, 34H); HRMS (ESI) m/z: $[M + H]^+$ calcd for $C_{65}H_{98}BrIN_{11}O_{20}$, 1558.5187; found, 1558.5212.

(((1S)-5-(N-(4-bromobenzyl)-2-((S)-45-(4-iodophenyl)-35,42-dioxo-36-(2-(4,7,10 tris(carboxymethyl)-1,4,7,10-tetraazacyclododecan-1-yl)acetamido) 4,7,10,13,16,19,22,25,28,31-decaoxa-34,41-diazapentatetracontanamido)hexanamido)-1-carboxypentyl)carbamoyl)-L-glutamic acid (^{177}Lu-Alb-L6): To the stirred solution of amine **14**

(10 mg, 0.0062 mmol, 1.0 eq) and IPBA-NHS (2.9 mg, 0.0074 mmol, 1.2 eq) in DMSO (100 μL) was added DIPEA (10.8 μL, 0.062 mmol, 10.0 eq) at room temperature. The reaction mixture was stirred for 2 h, and crude was diluted with water and purified by preparative RP-HPLC chromatography using 0.1% TFA in H_2O and 0.1% TFA in acetonitrile as eluents followed by lyophilization produced ^{177}**Lu-Alb-L6** (8 mg, 80%) as a white solid. [RP-HPLC purification was achieved using Agilent System, λ 220 nm, 250 mm × 10 mm Phenomenex Luna C18 column, solvent gradient: 90% H_2O (0.1% TFA) and 10% ACN (0.1% TFA), reaching 60% of ACN in 20 min at a flow rate of 10 mL/min. ^{1}H NMR (500 MHz, DMSO-d_6): δ 8.39 (s, 1H), 8.07 (s, 1H), 7.82 (t, *J* = 5.0 Hz, 1H), 7.67-7.73 (m, 2H), 7.42-7.50 (m, 2H), 7.07-7.13 (m, 4H), 6.99 (d, J = 10 Hz, 2H), 6.47 (d, 2H), 6.21-6.28 (m, 2H), 4.45 (s, 1H), 4.38 (s, 1H), 4.14-4.18 (m, 1H), 4.94-4.06 (m, 2H), 3.45-3.64 (m, 8H), 3.28-3.41 (m, 30H), 3.08-3.20 (m, 9H), 2.83-2.96 (m, 13H), 2.11-2.23 (m, 8H), 1.82-1.89 (m, 1H), 1.69-1.76 (m, 1H), 1.53-1.66 (m, 3H), 0.77-1.48 (m, 24H); HRMS (ESI) m/z: $[M + H]^+$ calcd for $C_{80}H_{130}BrIN_{11}O_{28}$, 1898.7305; found, 1898.7309.

HRMS (top) and ^{1}H NMR (bottom) spectra for ^{177}Lu-Alb-L1 in DMSO-d_6 at room temperature

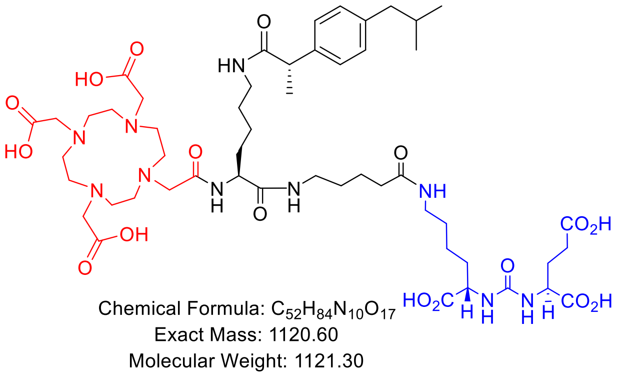

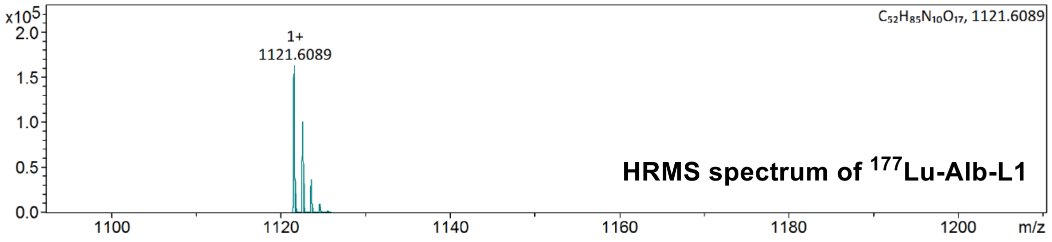

Meas. m/z	#	Ion Formula	m/z	err [ppm]	Mean err [ppm]	rdb	N-Rule	e^- Conf
1121.610088	1	C52H85N10O17	1121.608868	-1.1	0.5	15.5	ok	even

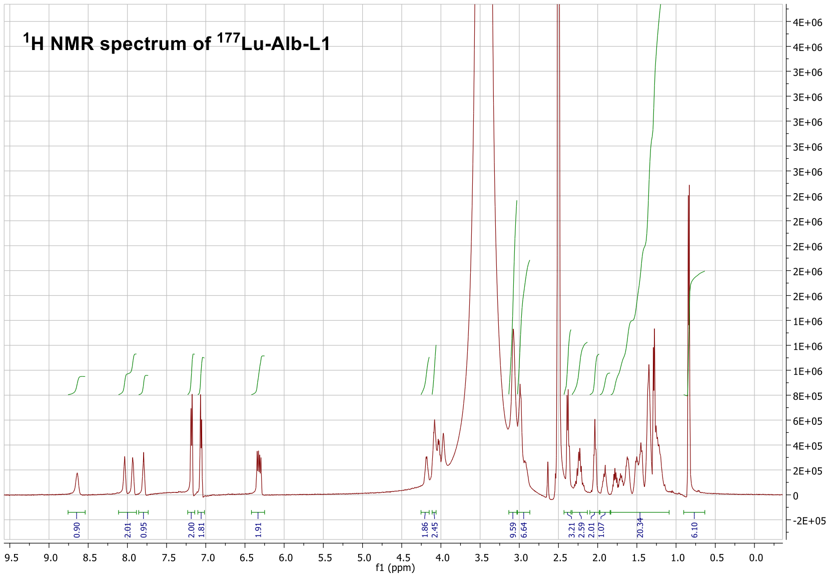

HRMS (top) and ^{1}H NMR (bottom) spectra for ^{177}Lu-Alb-L2 in DMSO-d_6 at room temperature

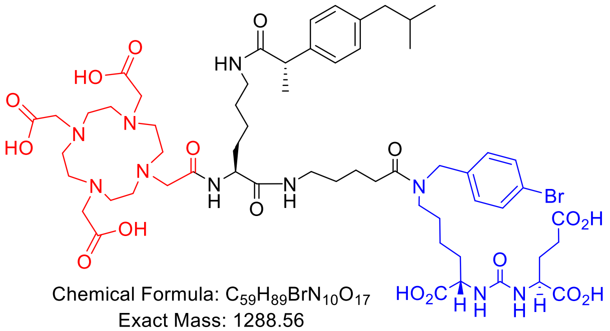

Chemical Formula: $C_{59}H_{89}BrN_{10}O_{17}$
Exact Mass: 1288.56
Molecular Weight: 1290.32

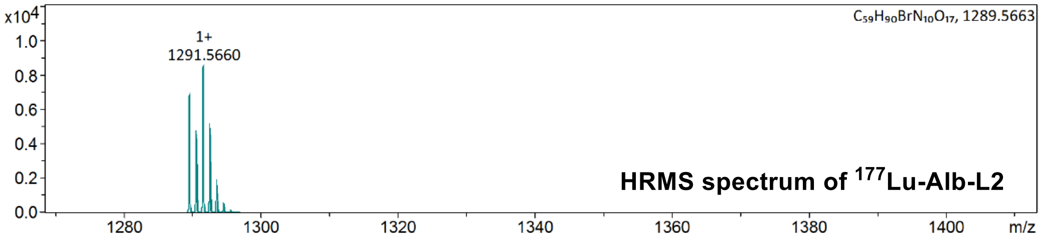

Meas. m/z	#	Ion Formula	m/z	err [ppm]	Mean err [ppm]	rdb	N-Rule	e⁻ Conf
1289.564272	1	C59H90BrN10O17	1289.566330	1.6	1.6	19.5	ok	even

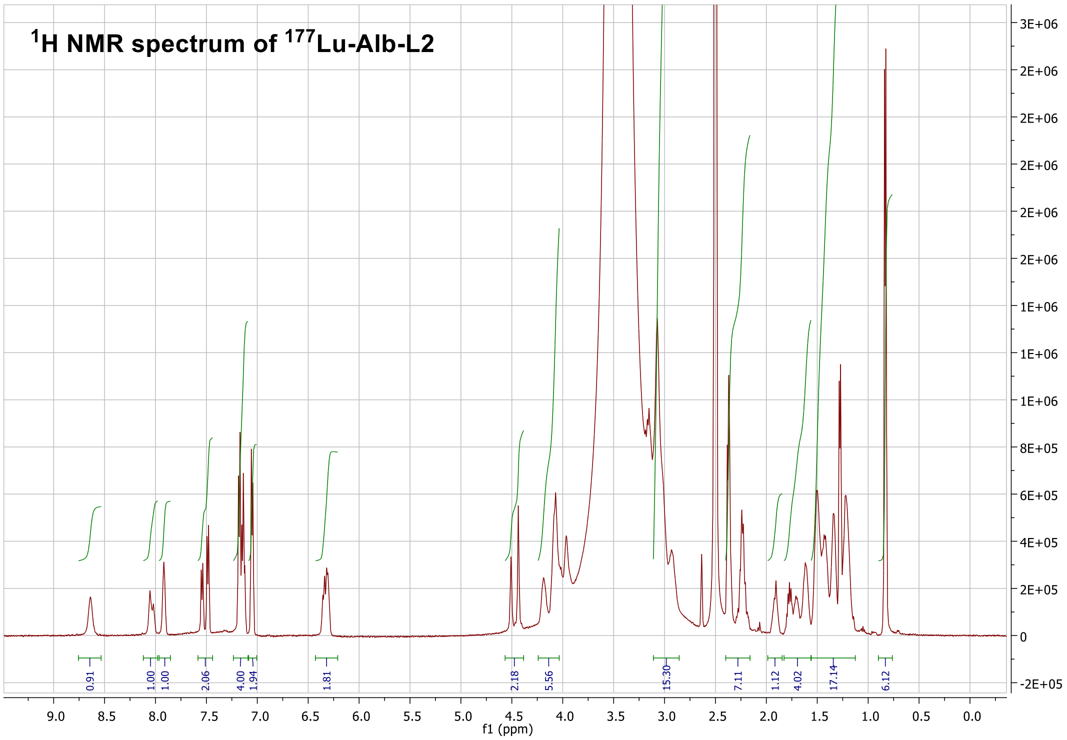

HRMS (top) and ^{1}H NMR (bottom) spectra for ^{177}Lu-Alb-L3 in DMSO-d$_6$ at room temperature

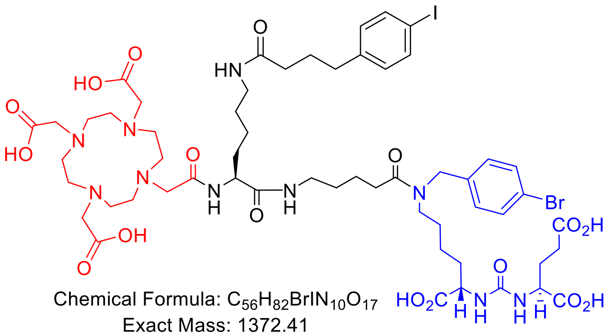

Chemical Formula: $C_{56}H_{82}BrIN_{10}O_{17}$
Exact Mass: 1372.41
Molecular Weight: 1374.13

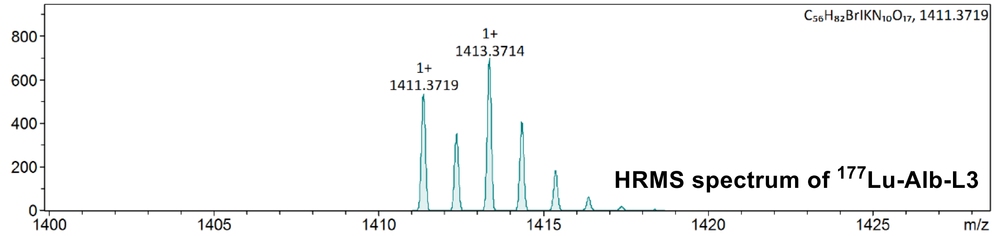

Meas. m/z	#	Ion Formula	m/z	err [ppm]	Mean err [ppm]	rdb	N-Rule	e⁻ Conf
1411.368895	1	C56H82BrIKN10O17	1411.371909	2.1	1441.4	19.5	ok	even

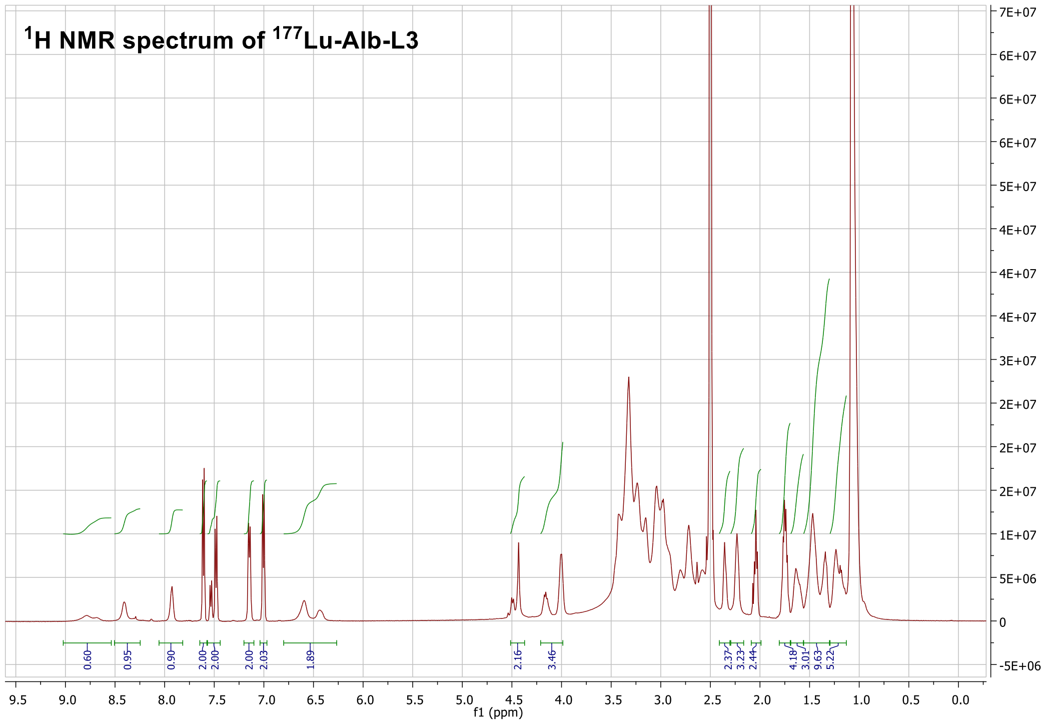

HRMS (top) and ^{1}H NMR (bottom) spectra for ^{177}Lu-Alb-L4 in DMSO-d$_6$ at room temperature

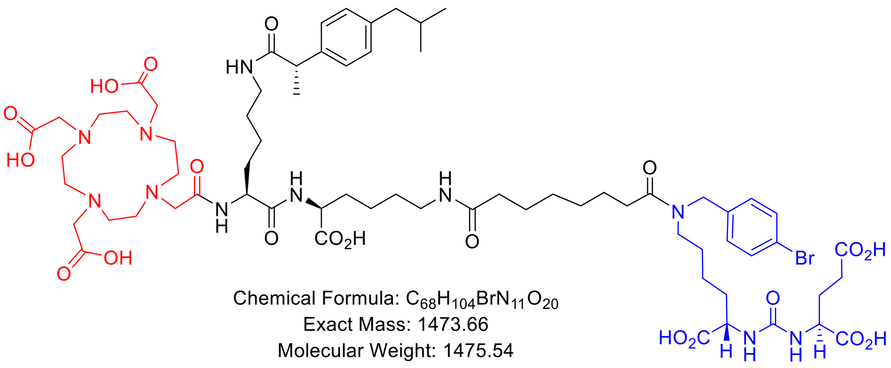

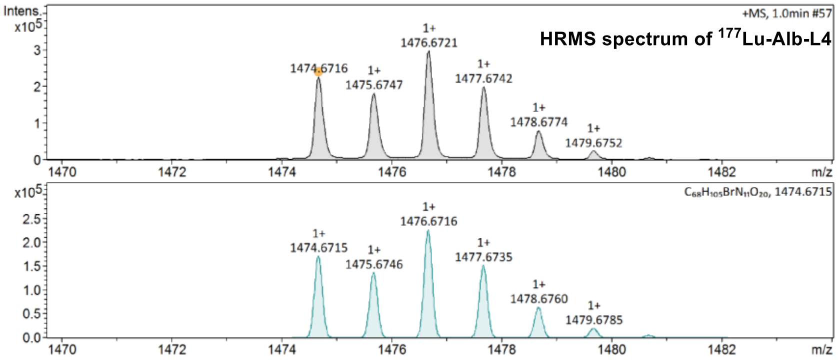

Meas. m/z	#	Ion Formula	m/z	err [ppm]	Mean err [ppm]	rdb	N-Rule	e⁻ Conf	mSigma	Std I	Std Mean m/z	Std I VarNorm	Std m/z Diff	Std Comb Dev
1474.671563	1	C68H105BrN11O20	1474.671523	-0.0	-0.0	21.5	ok	even	7.6	5.1	n.a.	n.a.	n.a.	n.a.

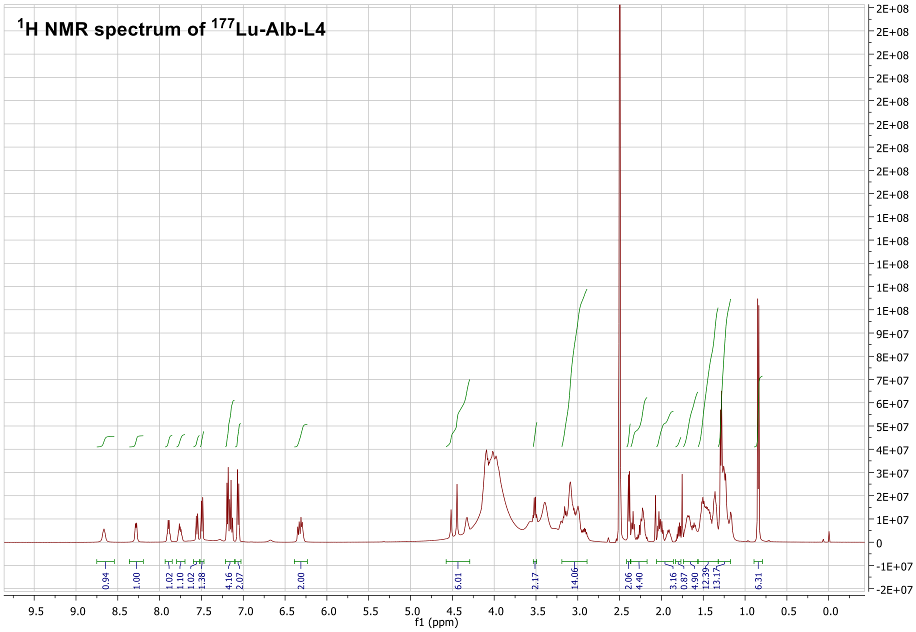

HRMS (top) and ^{1}H NMR (bottom) spectra for ^{177}Lu-Alb-L5 in DMSO-d$_6$ at room temperature

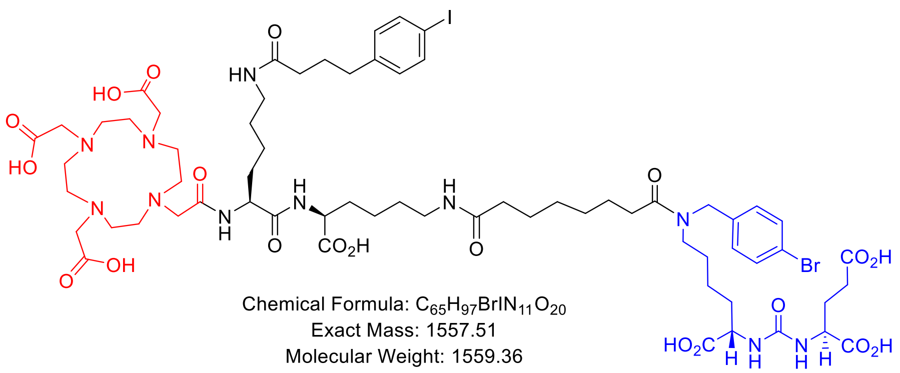

Chemical Formula: $C_{65}H_{97}BrIN_{11}O_{20}$
Exact Mass: 1557.51
Molecular Weight: 1559.36

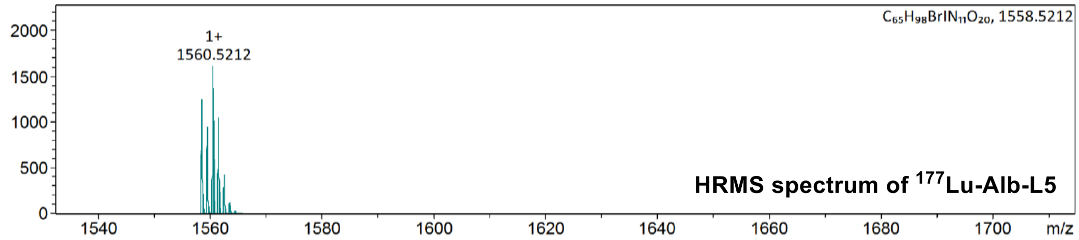

Meas. m/z	#	Ion Formula	m/z	err [ppm]	Mean err [ppm]	rdb	N-Rule	e⁻ Conf
1558.518742	1	C65H98BrIN11O20	1558.521221	1.6	425.3	21.5	ok	even

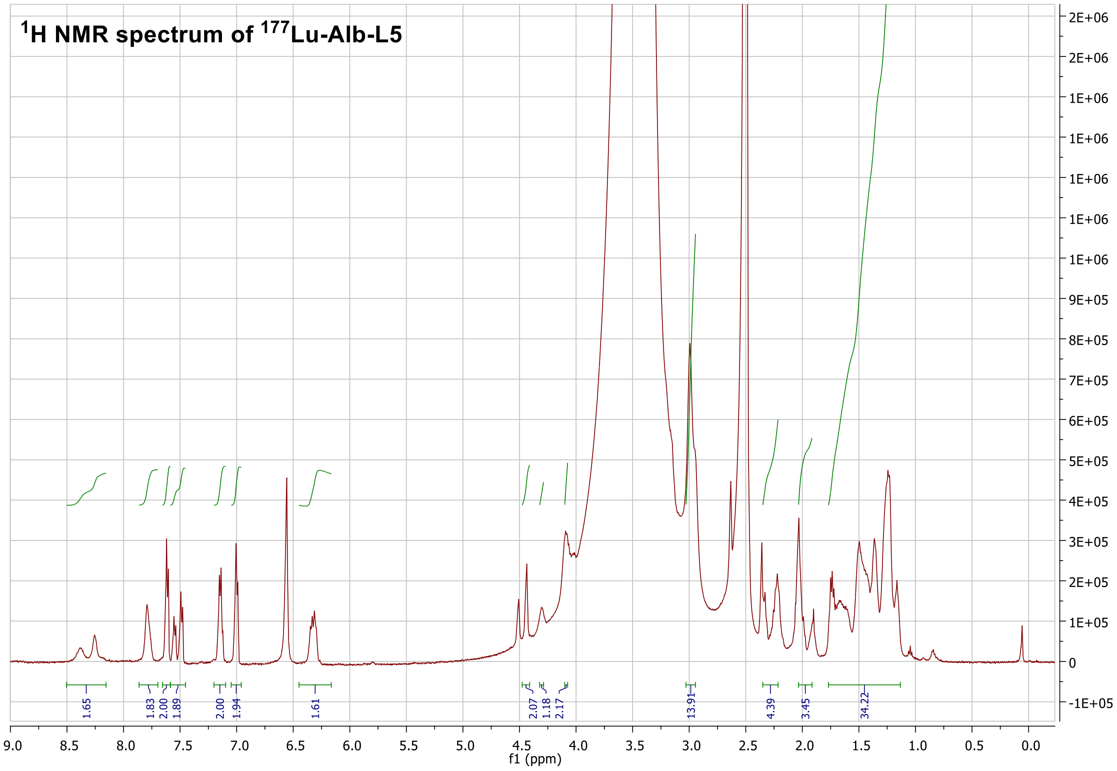

HRMS (top) and ^{1}H NMR (bottom) spectra for ^{177}Lu-Alb-L6 in DMSO-d_6 at room temperature

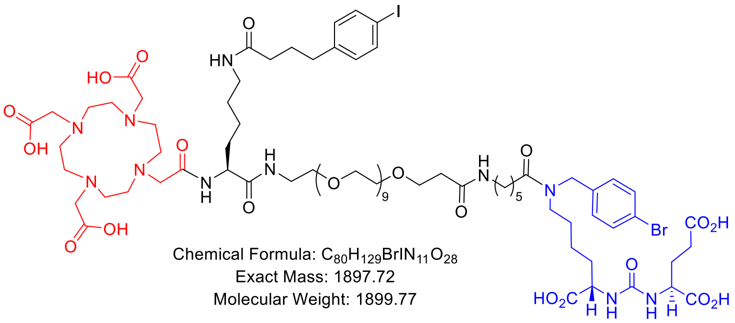

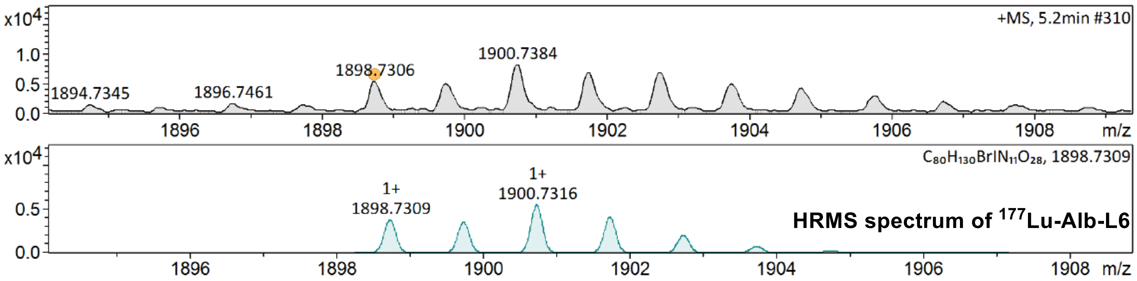

Meas. m/z	#	Ion Formula	m/z	err [ppm]	Mean err [ppm]	rdb	N-Rule	e⁻ Conf
1898.730567	1	C80H130BrIN11O28	1898.730939	0.2	264.8	20.5	ok	even

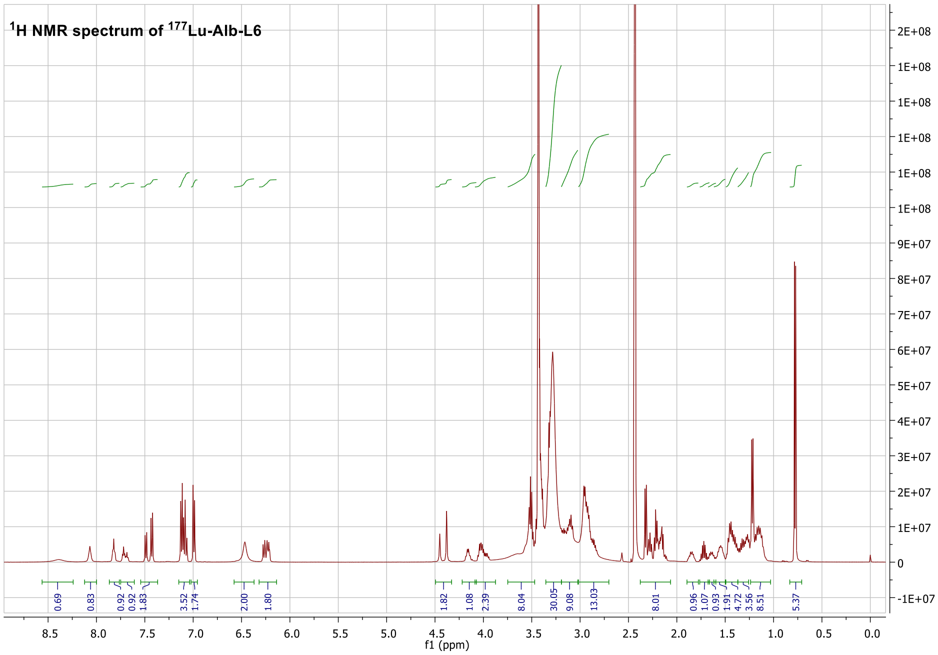

Representative HPLC data

HPLC chromatograms of ^{177}**Lu-Alb-L1**; radioactive peak (top), peak at λ = 220 nm (bottom). UV peak at 16.2-17.4 min is related to the unbound ligand **Alb-L1**.

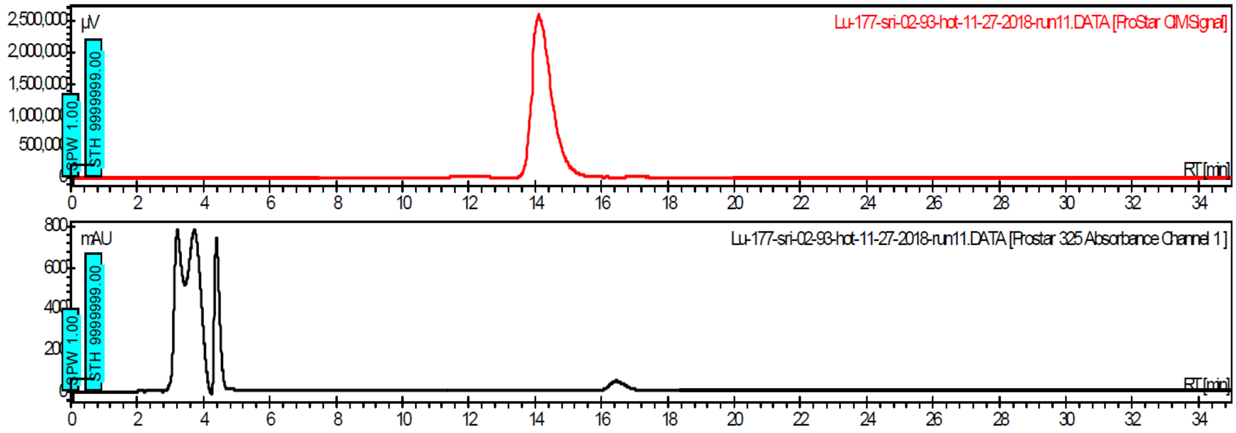

HPLC chromatograms of ^{177}**Lu-Alb-L2**; radioactive peak (top), peak at λ = 220 nm (bottom). UV peak at 36.2-38.4 min is related to the unbound ligand **Alb-L2.**

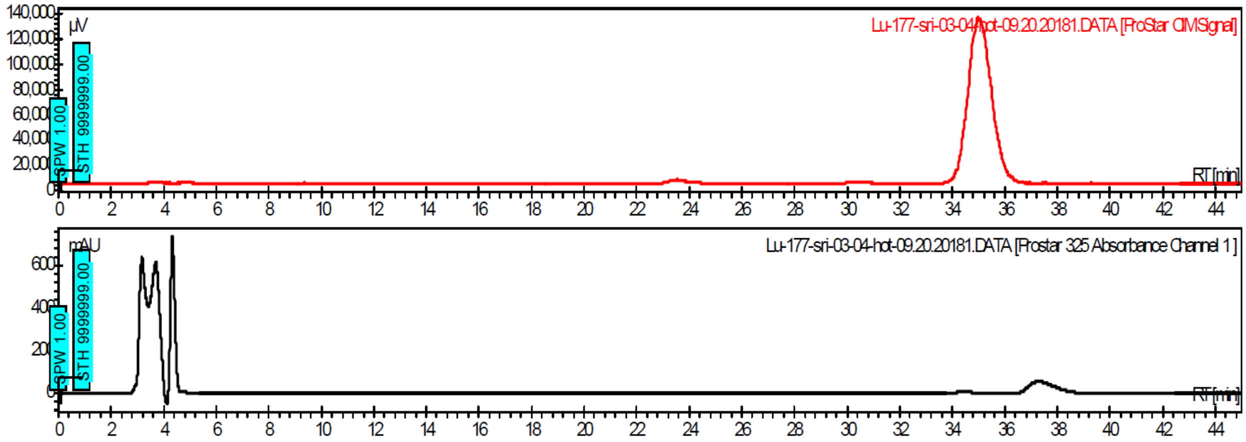

HPLC chromatograms of ^{177}**Lu-Alb-L3**; radioactive peak (top), peak at λ = 220 nm (bottom). UV peak at 32.4-33.2 min is related to the unbound ligand **Alb-L3**.

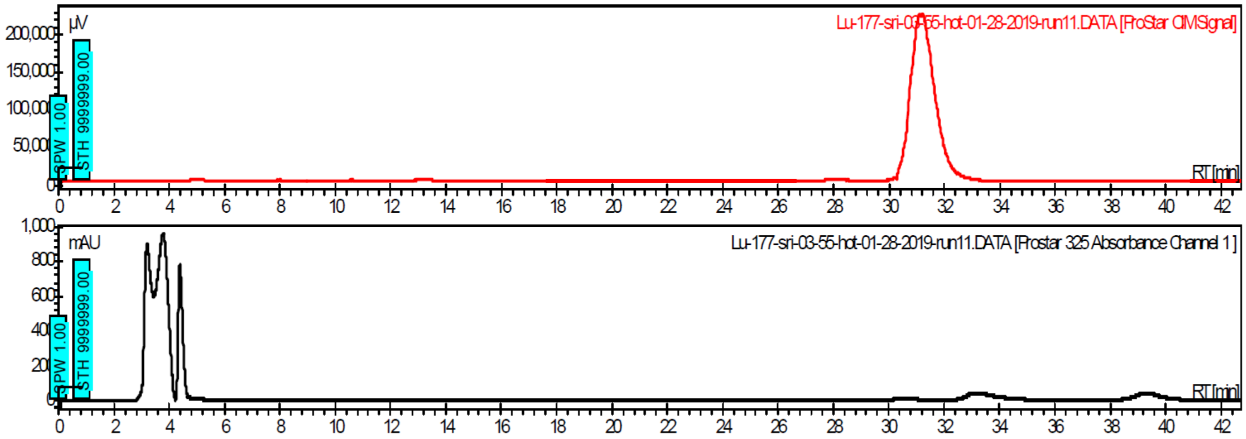

HPLC chromatograms of ^{177}**Lu-Alb-L4**; radioactive peak (top), peak at λ = 220 nm (bottom). UV peak at 38-39 min is related to the unbound ligand **Alb-L4**.

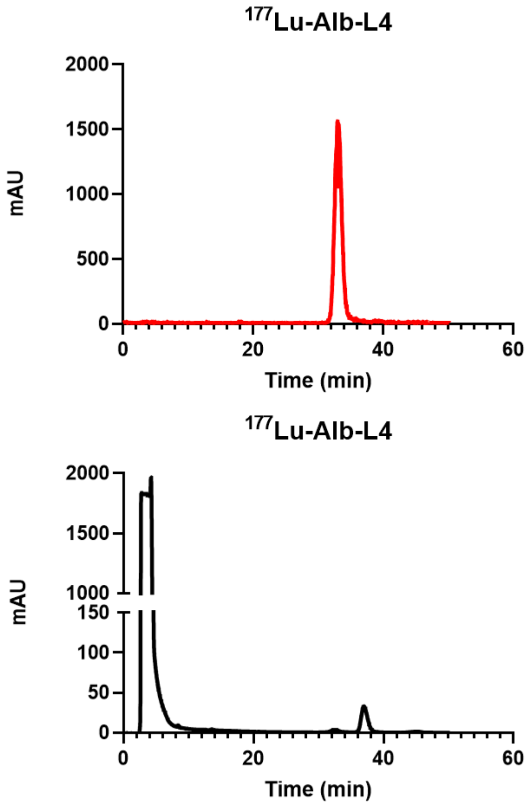

HPLC chromatograms of ^{177}**Lu-Alb-L5**; radioactive peak (top), peak at λ = 220 nm (bottom). UV peak at 41.0-43.0 min is related to the unbound ligand **Alb-L5**.

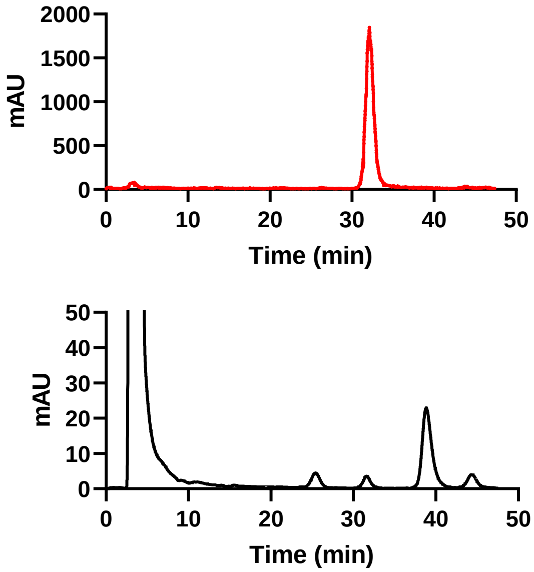

HPLC chromatograms of ^{177}**Lu-Alb-L6**; radioactive peak (top), peak at λ = 220 nm (bottom). UV peak at 58.0-61.0 min is related to the unbound ligand **Alb-L6**.

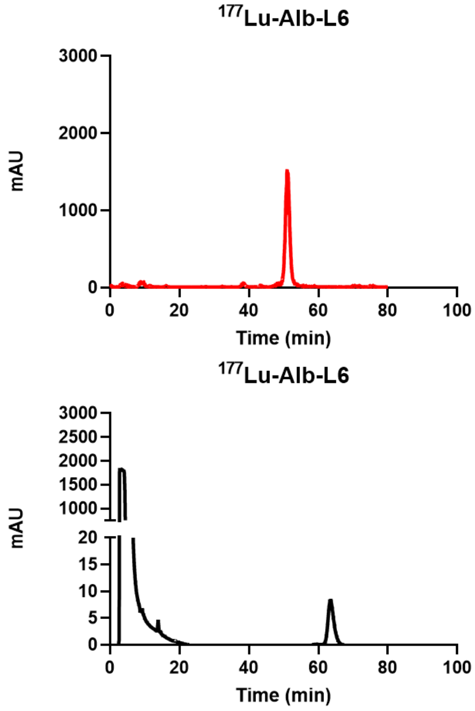

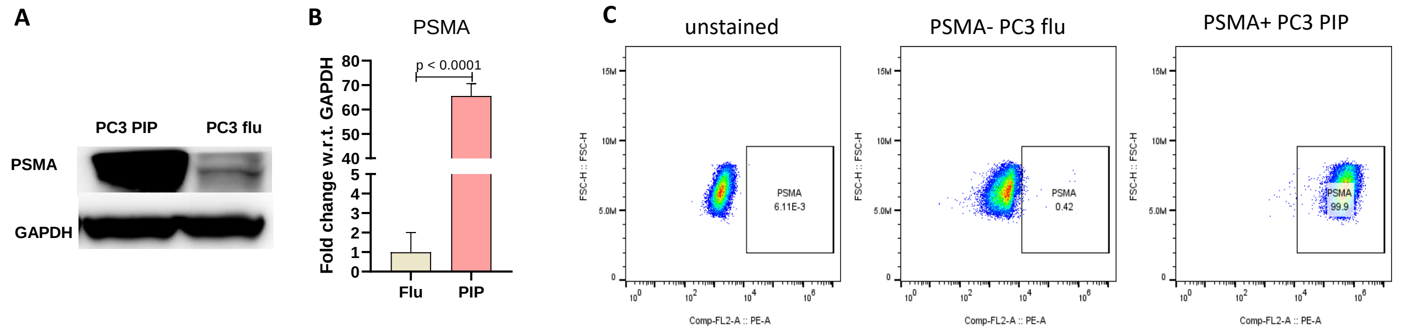

Supplementary Figure S1. Validation of PSMA expression in isogenic human prostate cancer PC3 sublines, PSMA+ PC3 PIP and PSMA- PC3 flu. PSMA+ PC3 PIP is designed to overexpress copious amounts of PSMA. **A.** PSMA total protein levels in PSMA+ PC3 PIP and PSMA- PC3 flu cells by western blot analysis. **B.** Real time PCR PSMA gene expression in PSMA+ PC3 PIP and PSMA- PC3 flu cells. **C**. Flow cytometry data showing PSMA surface expression on PSMA+ PC3 PIP and PSMA- flu cells.

Western Blot

PSMA+ PC3 PIP and PSMA- PC3 flu were homogenized on ice using radioimmunoprecipitation assay buffer (Sigma-Aldrich) containing protease inhibitor cocktail and subsequently sonicated to obtain a clear lysate. After centrifugation (10,000g for 15 min at 4°C) to remove cell debris, the supernatant was quantified using the protein assay kit bicinchoninic acid (Thermo Fisher Scientific). About 20 µg of each sample was separated and transferred onto a nitrocellulose membrane. Membranes were incubated in blocking buffer (2.5% BSA, 20% tween 20 in PBS) for 2 h, then washed with PBST and further incubated for 6 h with PSMA (cat#D718E, Cell Signaling Technology) and GAPDH (cat#D16H1, Cell Signaling Technology) antibodies at room temperature. Finally, membranes were incubated with HRP-coupled anti-rabbit IgG secondary antibodies, and blots were developed by ECL reagent. Digital quantification of chemiluminescence was performed using Image J software (NIH).

Real-Time PCR (RT-qPCR)

Cells were cultured to 80% confluence for mRNA isolation in 6 well-cell culture plates. cDNA was synthesized from 1 µg of total RNA of each experimental replicate using a cDNA synthesis kit (Applied Biosystems, USA) as per the manufacturer's protocol. The mRNAs were amplified on Applied Biosystems 7500 Fast Detection system with SYBR green qPCR master mix per manufacturer's instructions (Applied Biosystems, USA). All reactions were performed in triplicate, and negative controls were included in each experiment. GAPDH was taken as internal control,

and all the data sets were normalized to the level of GAPDH. Fold change in gene expression was calculated by the Δ2CT method, and results were reported as arbitrary units or fold changes.

Flow Cytometry

PSMA+ PC3 PIP and PSMA- PC3 flu cells (1 × 10^6 cells) were harvested using cell dissociation buffer (Gibco) and converted into a single-cell suspension. The harvested cells were washed twice with flow cytometry buffer (×1 phosphate-buffered saline with 2 mM ethylenediaminetetraacetic acid and 0.5% fetal bovine serum) and passed by pipetting through a 70 µm strainer. Next, the cells were stained with PSMA-PE antibody (cat#342504BioLegend,) according to the manufactured protocol. Cells were incubated at 4°C for 1 h in the dark. After 1 h, cells were washed with cold phosphate-buffered saline. The unstained and stained cells' fluorescence intensities were analyzed using flow cytometry (Attune NXT). Data were analyzed quantitatively by FlowJo software.

Supplementary Table S1. List of chemicals and solvents used in this report.

Manufacturer	Reagent Name
Chem-Impex	L-Glutamic acid di-tert-butyl ester hydrochloride, Nε-Z-L-lysine tert-butyl ester hydrochloride, Boc-5-aminovaleric acid, Trifluoroacetic acid, Nα-Fmoc-Nε-Boc-L-lysine N-hydroxysuccinimide ester, 2,5-dioxopyrrolidin-1-yl 5-((tert-butoxycarbonyl)amino)pentanoate, 2,5-dioxopyrrolidin-1-yl 6-((tert-butoxycarbonyl)amino)hexanoate, Fmoc-L-Lys(Boc)-OSu, O-(7-Azabenzotriazol-1-yl)-N,N,N',N'-tetramethyluronium hexafluorophosphate, 4-Pentynoic acid (HATU)
Millipore-SIGMA	Triphosgene, Triethylamine, Palladium on carbon, 4-Bromobenzaldehyde, Ibuprofen, Sodium triacetoxyborohydride, N-Hydroxysuccinimide, Piperidine, N, N-Diisopropylethylamine, 2% hydrazine-hydrate, N, N-Dimethylformamide anhydrous, Dimethyl sulfoxide, Dichloromethane anhydrous.
Broadpharm	t-Boc-N-amido-PEG10-NHS ester
Combi-Blocks	4-(p-Iodophenyl)butyric acid
CheMatech	DOTA-NHS-ester, disuccinimidyl suberate, DOTA-L-Lys(Dde)-L-Lys-NH_2
ThermoFisher Scientific	Ethyl acetate, Hexane, Acetonitrile anhydrous

Supplemental Table S2. HPLC Methods for ^{177}Lu-Alb-L1-^{177}Lu-Alb-L6

Ligand	HPLC Retention time	%A (0.1% TFA in Water)	%B (0.1% TFA in ACN)
^{177}Lu-Alb-L1	13.5-15.5 min	69	31
^{177}Lu-Alb-L2	34-36 min	64	36
^{177}Lu-Alb-L3	30-33.8 min	66	34
^{177}Lu-Alb-L4	36-40 min	64	36
^{177}Lu-Alb-L5	35.5-39 min	66	34
^{177}Lu-Alb-L6	35-40 min	66	34

Supplemental Table S3. Partition coefficient ($P_{Octanol}/_{water}$) and albumin binding properties of ^{177}Lu-Alb-L2-^{177}Lu-Alb-L6.

Ligand	Partition Co-efficient $P_{Octanol}/_{water}$	HSA (45 mg/mL)
^{177}Lu-L1	-2.47±0.01	29.4
^{177}Lu-Alb-L1	ND	ND
^{177}Lu-Alb-L2	-1.43±0.35	86.31%
^{177}Lu-Alb-L3	-1.74±0.20	79.89%
^{177}Lu-Alb-L4	-1.90±0.08	84.31%
^{177}Lu-Alb-L5	-0.81±0.31	91.29%
^{177}Lu-Alb-L6	-2.31±0.61	66.67%

Supplemental Table S4. Tissue biodistribution data of ^{177}Lu-Alb-L2 in male NSG mice bearing PSMA+ PC3 PIP and PSMA- PC3 flu xenografts in either flank (Data presented in % ID/g, expressed as mean ± SD) (n = 4).

**Time**	**2 h**	**24 h**	**48 h**	**192 h**
**Blood**	**19.59 ± 6.06**	**6.32 ± 1.37**	**2.90 ± 0.71**	**0.02 ± 0.01**
Heart	5.47 ± 1.22	1.75 ± 0.47	0.90 ± 0.27	0.01 ± 0.01
Lung	11.72 ± 3.15	4.39 ± 0.83	2.65 ± 0.65	0.03 ± 0.01
Liver	5.82 ± 1.81	1.46 ± 0.42	0.89 ± 0.35	0.02 ± 0.01
Stomach	1.97 ± 0.78	1.33 ± 0.24	0.84 ± 0.20	0.01 ± 0.00
Pancreas	2.32 ± 0.80	0.68 ± 0.15	0.38 ± 0.11	0.01 ± 0.00
Spleen	4.25 ± 1.00	1.58 ± 0.42	1.01 ± 0.34	0.02 ± 0.01
Fat	1.71 ± 0.40	0.64 ± 0.31	0.44 ± 0.23	0.04 ± 0.07
**Kidney**	**17.86 ± 5.09**	**7.44 ± 2.64**	**3.74 ± 1.05**	**0.05 ± 0.03**
Muscle	1.80 ± 0.63	0.57 ± 0.16	0.28 ± 0.04	0.00 ± 0.00
Small intestine	3.42 ± 1.66	1.73 ± 0.24	0.65 ± 0.27	0.00 ± 0.00
**Salivary. Gland**	**4.54 ± 1.49**	**1.60 ± 0.36**	**0.75 ± 0.20**	**0.02 ± 0.01**
Lacrimal gland	5.14 ± 1.28	1.94 ± 0.42	0.91 ± 0.20	0.04 ± 0.02
Bladder	3.51 ± 1.02	1.61 ± 0.29	0.67 ± 0.09	0.01 ± 0.00
Bone	2.52 ± 0.83	0.89 ± 0.17	0.52 ± 0.11	0.05 ± 0.01
**PSMA+ PC3 PIP**	**17.45 ± 6.51**	**26.41 ± 6.73**	**22.00 ± 4.71**	**3.39 ± 1.03**
PSMA- PC3 flu	2.68 ± 0.69	1.56 ± 0.27	0.87 ± 0.17	0.07 ± 0.02

Supplemental Table S5. Tissue biodistribution data of ^{177}Lu-Alb-L3 in male NSG mice bearing PSMA+ PC3 PIP and PSMA- PC3 flu xenografts in either flank (Data presented in % ID/g, expressed as mean ± SD) (n = 4).

Time	2 h	24 h	48 h	192 h
**Blood**	**10.29 ± 0.41**	**0.24 ± 0.12**	**0.10 ± 0.06**	**0.01 ± 0.02**
Heart	3.07 ± 0.27	0.09 ± 0.04	0.03 ± 0.008	0.004 ± 0.00
Lung	6.61 ± 0.63	0.29 ± 0.18	0.10 ± 0.01	0.035 ± 0.03
Liver	3.12 ± 0.91	0.18 ± 0.05	0.09 ± 0.01	0.033 ± 0.01
Stomach	1.35 ± 0.11	0.12 ± 0.05	0.05 ± 0.01	0.02 ± 0.01
Pancreas	1.46 ± 0.08	0.04 ± 0.02	0.02 ± 0.009	0.003 ± 0.00
Spleen	4.98 ± 0.66	0.27 ± 0.08	0.11 ± 0.04	0.02 ± 0.02
Fat	1.24 ± 0.26	0.24 ± 0.08	0.25 ± 0.12	0.08 ± 0.11
**Kidney**	**73.28 ± 22.85**	**2.77 ± 1.56**	**1.44 ± 0.76**	**0.27 ± 0.03**
Muscle	1.11 ± 0.35	0.04 ± 0.02	0.01 ± 0.007	0.002 ± 0.00
Small intestine	2.53 ± 0.43	0.12 ± 0.06	0.04 ± 0.006	0.004 ± 0.0
**Salivary gland**	**2.45 ± 0.20**	**0.10 ± 0.04**	**0.06 ± 0.02**	**0.011 ± 0.001**
Lacrimal gland	6.23 ± 4.81	0.16 ± 0.11	0.06 ± 0.03	0.04 ± 0.06
Bladder	7.66 ± 9.10	0.45 ± 0.38	0.12 ± 0.04	0.008 ± 0.01
Bone	1.62 ± 0.38	0.12 ± 0.03	0.08 ± 0.01	0.06 ± 0.01
**PSMA+ PC3 PIP**	**34.05 ± 1.98**	**30.55 ± 7.44**	**19.61 ± 4.46**	**5.94 ± 1.38**
PSMA+ PC3 flu	1.95 ± 0.10	0.33 ± 0.07	0.14 ± 0.02	0.04 ± 0.008

Supplemental Table S6. Tissue biodistribution data of ^{177}Lu-Alb-L4 in male NSG mice bearing PSMA+ PC3 PIP and PSMA- PC3 flu xenografts in either flank (Data presented in % ID/g, expressed as mean ± SD) (n = 4).

Time	2 h	24 h	48 h	192 h
**Blood**	**15.48 ± 7.12**	**2.19 ± 0.46**	**1.35 ± 0.12**	**0.36 ± 0.08**
Heart	5.84 ± 0.20	0.70 ± 0.13	0.48 ± 0.05	0.12 ± 0.01
Lung	10.61 ± 0.83	1.90 ± 0.35	1.23 ± 0.20	0.36 ± 0.06
Liver	6.47 ± 0.64	0.93 ± 0.19	0.57 ± 0.08	0.21 ± 0.05
Stomach	1.86 ± 0.24	0.36 ± 0.06	0.28 ± 0.06	0.11 ± 0.02
Pancreas	2.57 ± 1.56	0.32 ± 0.10	0.25 ± 0.09	0.09 ± 0.05
Spleen	17.98 ± 1.51	1.95 ± 0.35	1.41 ± 0.18	0.21 ± 0.09
Fat	2.15 ± 0.49	0.99 ± 0.59	1.22 ± 0.76	1.08 ± 0.63
**Kidney**	**64.44 ± 8.75**	**48.81 ± 19.86**	**23.46 ± 7.75**	**1.34 ± 0.82**
Muscle	1.23 ± 0.29	0.33 ± 0.20	0.18 ± 0.04	0.03 ± 0.02
Small intestine	4.92 ± 1.19	0.58 ± 0.16	0.41 ± 0.11	0.06 ± 0.03
**Salivary gland**	**5.40 ± 0.33**	**0.86 ± 0.21**	**0.62 ± 0.07**	**0.18 ± 0.06**
Lacrimal gland	7.92 ± 1.45	1.18 ± 0.30	1.10 ± 0.53	0.36 ± 0.27
Bladder	3.51 ± 0.10	1.38 ± 0.57	0.77 ± 0.47	0.39 ± 0.51
Bone	1.97 ± 0.43	0.50 ± 0.11	0.33 ± 0.03	0.06 ± 0.01
**PSMA+ PC3 PIP**	**40.89 ± 4.73**	**88.04 ± 16.51**	**87.74 ± 14.09**	**42.22 ± 14.05**
PSMA- PC3 flu	2.89 ± 0.79	1.17 ± 0.16	1.03 ± 0.15	0.33 ± 0.03

Supplemental Table S7. Tissue biodistribution data of ^{177}Lu-Alb-L5 in male NSG mice bearing PSMA+ PC3 PIP and PSMA- PC3 flu xenografts in either flank (Data presented in % ID/g, expressed as mean ± SD) (n = 4).

Time	2 h	24 h	48 h	192 h
**Blood**	**22.36 ± 4.72**	**11.67 ± 1.97**	**7.79 ± 0.81**	**2.77 ± 0.19**
Heart	7.10 ± 1.23	3.83 ± 0.50	2.92 ± 0.94	1.00 ± 0.12
Lung	14.67 ± 4.19	12.39 ± 1.40	7.53 ± 1.85	2.35 ± 0.18
Liver	9.23 ± 1.98	3.05 ± 0.74	1.94 ± 0.20	0.70 ± 0.11
Stomach	2.67 ± 0.67	2.20 ± 0.45	1.47 ± 0.14	0.42 ± 0.07
Pancreas	2.88 ± 0.70	1.71 ± 0.41	1.12 ± 0.14	0.47 ± 0.05
Spleen	16.34 ± 6.74	7.49 ± 1.42	5.31 ± 1.11	1.93 ± 0.25
Fat	3.44 ± 0.44	3.93 ± 2.06	1.97 ± 1.30	0.46 ± 0.11
**Kidney**	**33.45 ± 6.41**	**70.23 ± 16.64**	**48.17 ± 15.62**	**5.06 ± 0.99**
Muscle	1.65 ± 0.36	1.19 ± 0.33	0.91 ± 0.07	0.27 ± 0.03
Small intestine	3.97 ± 1.60	2.83 ± 0.46	1.92 ± 0.49	0.81 ± 0.23
**Salivary glands**	**5.83 ± 1.06**	**4.35 ± 0.66**	**3.20 ± 0.24**	**1.18 ± 0.09**
Lacrimal glands	7.33 ± 1.03	5.97 ± 0.51	4.44 ± 0.60	1.27 ± 0.14
Bladder	4.93 ± 2.61	4.25 ± 2.71	2.76 ± 0.29	1.04 ± 0.11
Bone	2.73 ± 0.61	3.35 ± 2.23	1.36 ± 0.32	0.53 ± 0.09
**PSMA+ PC3 PIP**	**28.78 ± 7.25**	**111.47 ± 13.85**	**127.44 ± 22.85**	**70.96 ± 2.34**
PSMA- PC3 flu	3.87 ± 0.70	4.55 ± 0.76	3.95 ± 0.50	1.87 ± 0.23

Supplemental Table S8. Tissue biodistribution data of ^{177}Lu-Alb-L6 in male NSG mice bearing PSMA+ PC3 PIP xenografts (Data presented in %ID/g, expressed as mean ± SD) (n = 4).

Time	2 h	24 h	48 h	192 h
**Blood**	**2.97 ± 0.45**	**0.03 ± 0.01**	**0.01 ± 0.01**	**0.01 ± 0.01**
Heart	0.95 ± 0.11	0.01 ± 0.01	0.01 ± 0.00	0.02 ± 0.01
Lung	5.46 ± 1.50	0.16 ± 0.11	0.29 ± 0.38	0.02 ± 0.00
Liver	1.22 ± 0.14	0.07 ± 0.01	0.04 ± 0.00	0.02 ± 0.01
Stomach	2.20 ± 0.56	0.05 ± 0.02	0.03 ± 0.01	0.05 ± 0.02
Pancreas	1.14 ± 0.76	0.01 ± 0.00	0.00 ± 0.00	0.01 ± 0.01
Spleen	1.68 ± 0.24	0.04 ± 0.03	0.02 ± 0.01	0.05 ± 0.02
Fat	0.54 ± 0.16	0.01 ± 0.01	0.01 ± 0.01	0.03 ± 0.02
**Kidney**	**18.26 ± 1.41**	**0.23 ± 0.08**	**0.09 ± 0.02**	**0.02 ± 0.00**
Muscle	0.45 ± 0.08	0.00 ± 0.00	0.00 ± 0.00	0.01 ± 0.00
Small Intestine	1.13 ± 0.12	0.03 ± 0.01	0.01 ± 0.00	0.01 ± 0.02
**Salivary Gland**	**1.05 ± 0.08**	**0.01 ± 0.00**	**0.01 ± 0.00**	**0.01 ± 0.01**
Lacrimal	1.36 ± 0.18	0.03 ± 0.02	0.02 ± 0.01	0.03 ± 0.03
Bladder	8.01 ± 1.88	0.11 ± 0.06	0.06 ± 0.04	0.09 ± 0.04
Bone	0.58 ± 0.05	0.02 ± 0.00	0.02 ± 0.00	0.02 ± 0.1
**PSMA+ PC3 PIP**	**38.73 ± 1.26**	**13.72 ± 3.55**	**9.62 ± 0.96**	**2.22 ± 0.37**

Supplemental Table S9. PSMA+ PC PIP tumor weight (g) and expressed as mean±SD) and tumor volume (mm^3, expressed as mean ±SD) used for biodistribution studies.

Ligand		2 h	24 h	48 h	8 d
^{177}Lu-Alb-L2	PIP weight	0.27±0.08	0.27±0.10	0.29±0.09	0.50±0.50
	Tumor size	1169.56±857.33	529.06±271.80	411.04±96.04	807.44±155.84
^{177}Lu-Alb-L3	PIP weight	0.36±0.09	0.40±0.06	0.28±0.04	0.41±0.13
	Tumor size	NA	NA	NA	NA
^{177}Lu-Alb-L4	PIP weight	0.22±0.03	0.39±0.22	0.48±0.23	0.57±0.35
	Tumor size	NA	NA	NA	NA
^{177}Lu-Alb-L5	PIP weight	0.17±0.06	0.22±0.02	0.21±0.07	0.36±0.07
	Tumor size	494.93±119.28	508.19±76.80	535.15±137.72	384.44±48.58
^{177}Lu-Alb-L6	PIP weight	0.26±0.07	0.09±0.06	0.16±0.06	0.31±0.13
	Tumor size	218.14±57.29	122.59±51.20	163.83±38.25	170.63±79.93

Supplemental Table S10. Tissue biodistribution data of ^{177}Lu-Alb-L1 in male NSG mice bearing PSMA+ PC3 PIP and PSMA- PC3 flu xenografts in either flank (Data presented in % ID/g, expressed as mean ± SD) (n = 3-4) at 24 h post-injection. The biodistribution studies of ^{177}Lu-Alb-L2, ^{177}Lu-Alb-L5, and ^{177}Lu-L1(A, 24 h) were done using the same batch of mice and the same radioactivity. The biodistribution studies of ^{177}Lu-Alb-L3, ^{177}Lu-Alb-L4, and ^{177}Lu-L1 (B, 24 h) were done head-to-head using the same batch of mice and radioactivity. The data are presented below.

Time	24 h (A)	24 h (B)
**Blood**	**0.01 ±0.01**	**0.00±0.00**
Heart	0.00±0.00	0.01±0.00
Lung	0.02±0.02	0.01±0.00
Liver	0.02±0.01	0.02±0.00
Stomach	0.01±0.00	0.01±0.00
Pancreas	0.00±0.00	0.00±0.00
Spleen	0.03±0.02	0.01±0.00
Fat	0.03±0.03	0.03±0.04
**Kidney**	**0.32±0.16**	**0.11±0.03**
Muscle	0.00±0.00	0.00±0.00
Small Intestine	0.01±0.00	0.01±0.00
Salivary Gland	0.01±0.01	0.01±0.00
Lacrimal	0.08±0.05	0.05±0.04
Bladder	0.02±0.02	0.02±0.00
Bone	0.03±0.00	0.04±0.01
**PSMA+ PC3 PIP**	**12.86±0.98**	**14.47±1.32**
PSMA+ PC3 flu	0.01±0.01	0.01±0.00

Reference cited

1. Banerjee, S. R., Kumar, V., Lisok, A., Chen, J., Minn, I., Brummet, M., Boinapally, S., Cole, M., Ngen, E., Wharram, B., Brayton, C., Hobbs, R. F., and Pomper, M. G. (2019) ^{177}Lu-labeled low-molecular-weight agents for PSMA-targeted radiopharmaceutical therapy. *Eur J Nucl Med Mol Imaging* **46**, 2545-2557
